# Insight on the impact of *Acacia nilotica* leaves extract on the characteristics of natural and synthetic rubber composites

**DOI:** 10.1038/s41598-026-40512-8

**Published:** 2026-03-19

**Authors:** T. A. Zidan, Sayed A. El-Toumy, M. N. Ismail, D. E. El-Nashar, A. I. Khalaf

**Affiliations:** 1 Polymers and Pigments Department, National Research Centre, Dokki, Giza, 12622 Egypt; 2 Chemistry of Tannins Department, National Research Centre, Dokki, Giza, 12622 Egypt

**Keywords:** Natural antioxidant, *Acacia nilotica*, Physico-mechanical properties, Aging properties, Rubber, Biochemistry, Chemistry, Materials science, Plant sciences

## Abstract

The purpose of this study is to assess the extract of *Acacia nilotica* leaves as a sustainable natural antioxidant for natural rubber (NR) and styrene-butadiene rubber (SBR) composites. The Acacia extract was incorporated to NR and SBR at concentrations from 0.5 to 2 phr, and its effectiveness was compared to that of commercial antioxidant polymerized 2,2,4-trimethyl-1,2-dihydroquinoline (TMQ). Rubber compositions were evaluated for chemical interactions and dispersion using FTIR and FESEM, and curing behavior was realized by rheometric studies. Physico-mechanical characteristics were assessed prior to and following thermo-oxidative aging at 90°Cfor different periods (2, 4, 6, and 7 days). The findings show that in both rubber matrices, theAcacia extract significantly improves aging resistance. While greater loadings (≥ 1 phr) negatively impacted initial strength without further improving aging resistance in comparison to TMQ. NR composites demonstrated an optimal antioxidant performance at 0.5 phr, offering superior retention of tensile strength during aging. At ideal concentrations of 0.5–1 phr, the extract dramatically increased tensile strength and elongation at break in SBR composites, exceeding TMQ in preventing excessive modulus expansion during aging. These results validate the potential of Acacia extractas an environmentally friendly antioxidant for rubber applications, especially in automotive, sealing, and vibration-damping components where improved durability and sustainable material solutions are needed.

**Supplementary Information:**

The online version contains supplementary material available at 10.1038/s41598-026-40512-8.

## Introduction

Natural and synthetic diene rubbers^1–5^ play crucial roles in a variety of industries, including tires and damping materials, because of their exceptional elasticity, strength, toughness, and strain performance^6–9^. As a result, rubber is recognized as a strategic commodity on a global scale^10,11^. Nevertheless, diene rubbers, especially those with double bonds, are very vulnerable to thermo-oxidative reactions when subjected to heat and oxygen^12,13^. This causes a major decline in its mechanical qualities, hardness, and attractiveness, which shortens its lifespan significantly^14–16^. Nonetheless, they are susceptible to thermal oxidation damage during storage and serving because of the high concentration of unsaturated carbon–carbon double bonds and reactive allylichydrogens present in their macromolecular structures^17^. Generally, the addition of chemical antioxidants seems to be the most convenient and effective ways to prevent or delay the thermo-oxidative aging of rubbers^18–20^. Amines and hindered phenolic antioxidants are the two most prevalent forms of antioxidants for rubber; they are both radical scavengers. By neutralizing free radicals through either a hydrogen atom transfer (HAT) or a single electron transfer (SET) reduction mechanism, these potent reducing agents stop free radicals from attacking the rubber structure^21,22^.

The most popular raw material for numerous industrial applications needing adequate mechanical strength, adhesive ability, and resilience is natural rubber (NR)^23,24^. Styrene butadiene rubber is one of the most popular synthetic rubbers due to its high fracture elongation. On the other hand, its durability and elastic modulus are low. In addition to other rubber products, it is used in the tire business. A high degree of unsaturation makes it possible for different vulcanization processes to effectively cross-link. But it also weakens the rubber’s resistance to oxidative and thermal degradation. Degradation may be accelerated by external causes such as humidity, harsh chemicals, mechanical loads, radiation, and corrosive pollutants^25–27^. So, antioxidants were added during mixing. Zidan T.A. and Yehia A.A. prepared derivatives from commercial antioxidant TMQ and evaluate the prepared compounds as antioxidants for SBR in comparison to the commercial TMQ. They observed that the prepared compounds have high antioxidant activity compared to TMQ^28^.

Natural compounds exhibit different applications^29–32^. Among these compounds are natural antioxidants. Because natural antioxidants are less expensive systems for all industrial areas under investigation, scientists nowadays tend to use them for environmental protection, even though synthetic antioxidants are quite effective at degrading polymers but cause temporary and/or permanent harm to the environment and human body^33,34^. Research from the past and present has focused a lot of emphasis on extracts from renewable plants. The antioxidant effects of certain amino acids and keratin derived from chicken feathers in radiation-vulcanized natural rubber latex were investigated by Abad et al.^35^. In order to stop NR composites from aging, Komethi et al.^36^ investigated the leaves of palm trees (Elaeisguineensis). The performance of this natural antioxidant was examined using a comparative methodology. Butylatedhydroxytoluene (BHT) and commercially available TMQ were regarded as competitors. The antioxidant activity of chitosan derivatives in composites based on acrylonitrile butadiene rubber (NBR) and NR were examined by Khalaf et al.^27^. In comparison to conventional TMQ, Zidan T.A. demonstrated high activity using rhodanine-chitosan hydrogel as an antioxidant for NBR composites^37^. By contrasting it with TMQ, Shuhaimi et al.^23^ examined the antioxidation capacity of palm tree oil for a range of NR vulcanization systems. Crude propolis was recently investigated by Kmiotek et al.^38^ for stabilizing peroxide-cured NR in the presence of microbes, oxygen, and ozone. Composites containing propolis were discovered to have very promising after-aging characteristics. Furthermore, the study’s most noteworthy finding was that propolis had long-lasting antibacterial and antifungal properties. In contrast to the most popular rubber antioxidants, TMQ and IPPD, Abdel Zaher et al.^39^ assessed the effectiveness of lignin/silica and calcium lignate/calcium silicate in preventing thermal aging of styrene-butadiene rubber (SBR). Thermo-oxidative degradation and peroxidation behavior of saturated (like isoprene rubber) and unsaturated (like butadiene rubber, and styrene-butadiene rubber) rubbers with 2,6-di-tert-butyl-4-methylphenol (BHT), vitamin A (b-carotene), and vitamin E (a-toco-pherol) were compared by Ivan et al.^40^. The effects of erucic and syringic acids on the antioxidant qualities of natural rubber were investigated by Shihao Chen et al.^41^. Henna leaf powder was assessed by öncel et al. as a natural substitute antioxidant for natural rubber^34^. Although plant-based antioxidants have been previously explored for rubber stabilization, the systematic application of *A. nilotica* leaf extract as a multifunctional antioxidant for rubber has not been comprehensively reported. The novelty of this work lies in the comparative evaluation of *Acacia nilotica* extract across rubber matrix under identical processing and aging conditions, using a commercial antioxidant (TMQ) as a direct benchmark. The findings demonstrate that *A. nilotica* leaf extract can function not only as an eco-friendly antioxidant but also as a tunable performance modifier, offering a viable and sustainable alternative to synthetic antioxidants for industrial rubber applications requiring durability and environmental compatibility. In this work, we areinterested in studying the antioxidant activity of the extract of *Acacia nilotica* leaves (A)in natural rubber (NR) and synthetic rubber (SBR) by comparing the A with the other commercial antioxidant TMQ. Surface morphology, rheometric characteristics, mechanical (before and after thermal aging), and swelling properties of NR and SBR compositesare evaluated. The obtained results are compared with that obtained using commercial antioxidant TMQ.

## Materials and experimental techniques

### Materials

Styrene butadiene rubber (SBR 1502 non-staining) with a styrene content of 23.5%, specific gravity of 0.945, Tg with − 60 °C, and Mooney viscosity M_L_ (1 + 4) of 52 ± 3 at 100 °C was supplied by the Transport and Engineering Company (TRENCO) in Alexandria. Natural rubber (NR) of type (RSS-1) with a density of 0.913 ± 0.005 g/cm^3^ at 23 °C, Tg = − 75 °C, and Mooney viscosity M_L_(1 + 4) at 100°C = 60–90 was acquired from Transport and Engineering, Alexandria, Egypt. At 15 °C, 0.90–0.97 specific gravity stearic acid and zinc oxide with a specific gravity of 5.55–5.61 were used as activators. Poly(2,2,4-trimethyl-1,2-dihydroquinoline) [TMQ], a commercial antioxidant, was used. Carbon black (high abrasion furnace; HAF) was used as a strengthening ingredient. Processing oil was the plasticizer used in the procedure. N-cyclohexyl-2-benzothiazole sulphenamide (CBS), an accelerator having a specific gravity of 1.27–1.31 at room temperature and a melting point of 95–100 °C, was used. Sulfur was used as an elemental vulcanizing agent. It is a fine, and paleyellow powder with a specific gravity of 2.04 to 2.06 at room temperature. Leaves of *Acacia nilotica* were collected from Upper Egypt. The extract was obtained and identified at the Chemistry of Tannins department, National Research Centre, Egypt.

### Extraction and isolation of compounds

The leaves of *A. nilotica* (1 kg) were defatted with CHCl_3_ (3 × 1 L) and extracted with CH_3_OH: H_2_O (7:3; 5 × 3 L) at room temperature. After filtering, the mixed extracts were lyophilized (60 g) and evaporated under low pressure. A polyamide 6S column chromatography (80 × 3 cm) was loaded with the dried extract. After eluting the column with H_2_O, ten fractions (1 L each) of H_2_O-EtOH mixtures with decreasing polarity were recovered. Following chromatographic examination, the main phenolic fractions were merged into four fractions. Fraction A (2.2 g) was fractionated by column chromatography on Sephadex LH-20 with aqueous EtOH (0- 70%) for elution to give compounds **1** and **3**. Fraction B (2.5 g) was subjected to column chromatography on cellulose and n-BuOH saturated with H_2_O as an eluent to give two major subfractions, and then each of them was separately fractionated on a Sephadex LH-20 to yield pure samples **6** and **5.** (Using the same procedure fraction C (2.8 g) and fraction D (2.4 g) gave chromatographically pure samples **4**, **2** and **7**.

Phytochemical investigation of *A. nilotica * resulted in the isolation of gallic acid **1**, methyl gallate **2**, catechin **3**, quercetin-3**-*O*-** rhanmnopyranosyl (1–6) glucopyranoside **4**, quercetin-3-***O***-α-L-rhamnopyranoside **5**, quercetin-3-***O***- β- glucopyranoside **6**, and quercetin **7**. The compounds were isolated using column Chromatography and the structures were determined with sample and spectroscopic techniques. Supplementary materials (S1-S6) illustrate the chemical structures, ^1^H NMR and ^13^C NMR of the isolated compounds, respectively. UV, ^1^H NMR and ^13^C NMR data of the isolated compounds are represented as follows:

**Gallic acid (1)**

**UV Spectral Data λ**_**max**_** (nm):** (+ MeOH) 272 nm.

^**1**^**H NMR (400 MHz, DMSO-d6) δ (ppm):**6.98 (2H, s, H-2/6.

^**13**^**C NMR (100 MHz, DMSO-d6) δ (ppm):** 167.7 (C = O), 145.5 (C-3/5), 138.1(C-4),120.6(C-1),108.8(C-2/6).

**Methyl Gallate, (2)**

**UV Spectral Data λ**_**max**_** (nm):**MeOH220, 275.

^**1**^**H NMR (400 MHz, DMSO-d6) δ (ppm):** 6.95 (s, H-2 and H-6), 3.72 (s, methylester protons).

^**13**^**C NMR (100 MHz, DMSO-d6) δ (ppm):** 119.80 (C-1), 108.80 (C-2), 144.20 (C-3), 139.10 (C-4),144.20 (C-5), 108.80 (C-6), 167.20 (C-7), 51.60 (COO-CH_3_).

**Catechin, (3):**

**UV Spectral Data λ**_**max**_** (nm):**MeOH (a): 230^sh^, 278.

^**1**^**H NMR (400 MHz, DMSO-d6) δ (ppm):**6.72 (d, J =1.4 Hz, H-2'), 6.69 (d, J =8.1 Hz, H-5'), 6.59 (dd, J = 8.1 Hz and J = 1.4 Hz, H-6'), 5.88 (d, J =2.1 Hz, H-6), 5.70 (d, J=2.1 Hz, H-8), 4.49 (d, J=7.34 Hz, H-2), 3.63 (m, H-3), 2.65 (eq., dd, J = 15.87 Hz and J = 5.31, H-4) or 2.35 (ax., dd, J = 15.94 Hz and J = 7.86 Hz, H-4).

^**13**^**C NMR (100 MHz, DMSO-d6) δ (ppm):** 81.27 (C-2), 66.63 (C-3), 28.09 (C-4), 156.50 (C-5), 95.50 (C-6), 156.76 (C-7), 94.24 (C-8), 155.67 (C-9), 99.45 (C-10), 130.94 (C-1'), 114.81 (C-2'), 145.16 (C-3'), 145.16 (C-4'), 115.46 (C-5'), 118.82 (C-6').

**Quercetin 3-** ***O*** **- α-L-rhamnopyranosyl- (1 → 6)-β-D-glucopyanoside (4)**

**UV Spectral Data, λ max (nm)**MeOH: 258 nm, 358 nm.

^**1**^**H NMR (400 MHz, DMSO-d6) δ (ppm):Aglycone**7.57 (1H, d, J = 2.1 Hz, H-2’), 7.54 (1H, dd, J = 9, 2.1 Hz, H-6’), 6.85 (1H, d, J = 9 Hz, H-5’), 6.39 (1H, d, J = 2.1 Hz, H-8), 6.2 (1H, d, J = 2.1 Hz, H-6).

**Sugar** 5.35 (1H, d, J = 7.5 Hz, H-1’’), 4.4 (1H, d, J = 2.0 Hz, H-1’’’), 3.16–3.65 (9H, m, H-2’’- H-6’’, H-2’’’- H-5’’’), 1.01 (3H, d, J = 6.3 Hz, CH_3_ of rhamnose**).**

^**13**^**C NMR (100 MHz, DMSO-d6) δ (ppm): Aglycone:** 158.6 (C-2), 135.6 (C-3), 179.4 (C-4), 163.0 (C-5), 100.1 (C-6) , 166.5 (C-7), 95.0 (C-8), 159.3 (C-9), 105.5 (C-10), 123.1 (C-1’), 117.7 (C-2’), 145.9 (C-3’), 149.9 (C-4’), 116.1 (C5’), 123.5 (C-6’).

**Sugar:** glucose: δ (ppm): 104.8 (C-1’’), 75.7 (C-2’’), 78.2 (C-3’’), 71.4 (C-4’’), 77.2 (C-5’’), 68.6 (C-6’’).

Rhamnose at 3- position as terminal sugar: δ (ppm): 102.4 (C-1’’’), 72.1(C-2’’’), 72.2 (C-3’’’), 73.9 (C-4’’’), 69.7 (C-5’’’), 17.9 CH3 of Rhamnose.

**Quercetin-3-** ***O*** **-α-L-rhamnopyranoside (5)**

**UV Spectral Data λ**_**max**_** (nm)**: MeOH: 253 nm, 263sh, 344 nm.

^**1**^**H NMR (400 MHz, DMSO-d6) δ (ppm): Aglycone** 7.33 (1H, d, J = 2.0 Hz, H-2’), 7.28 (1H, dd, J = 2.0 Hz, and J = 8.5 Hz, H-6’), 6.9 (1H, d, J = 8.5 Hz, H-5’), 6.42 (1H, d, J = 2.0 Hz, H-8), 6.23 (1H, d, J = 2.0 Hz, H-6).

**Sugar:** 5.29 (1H, d, J = 1.41 Hz, H-1’’), 3.16–3.94 (4H, m, H-2’’- H-5’’), 1.03 (3H, d, J = 6.15 Hz, CH_3_ of rhamnose).

^**13**^**C NMR (100 MHz, DMSO-d6) δ (ppm): Aglycone **157.34 (C-2), 134.27 (C-3), 177.79 (C-4), 161.35 (C-5), 98.78 (C-6), 164.34 (C-7), 93.70 (C-8), 156.86 (C-9), 104.16 (C-10), 121.17 (C-1’), 115.52 (C-2’), 145.25 (C-3’), 148.50 (C-4’), 115.72 (C-5’), 120.80 (C-6’).

**Sugar: δ (ppm):** 101.87 (C-1’’), 70.43 (C-2’’), 70.63 (C-3’’), 71.25 (C-4’’), 70.11 (C-5’’), 17.54 (C-6’’).

**Quercetin 3-** ***O*** **-β-D-glucopyranosyl (6)**

**UV Spectral Data, λ max (nm)** MeOH: 253 nm, 263sh, 294sh, 351 nm.

^**1**^**H NMR (400 MHz, DMSO-d6) δ (ppm): Aglycone** 7.67 (1H, dd, J = 2.12 Hz, and J = 8.62 Hz, H-6’), 7.53 (1H, d, J = 2.12 Hz, H-2’), 6.82 (1H, d, J = 8.62 Hz, H-5’), 6.4 (1H, d, J = 1.8 Hz, H-8), 6.2 (1H, d, J = 1.8 Hz, H-6).

**Sugar:** 5.37 (1H, d, J = 7.63 Hz, H-1’’), 3.28–3.65 (5H, m, H-2’’- H-6’’).

^**13**^**C NMR (100 MHz, DMSO-d6) δ (ppm): Aglycone** 156.80 (C-2), 133.60 (C-3), 177.50 (C-4), 161.60 (C-5), 98.90 (C-6), 164.60 (C-7), 93.80 (C-8), 156.60 (C-9), 104.00 (C-10), 121.60 (C-1’), 115.80 (C-2’), 145.80 (C-3’), 148.80 (C-4’), 116.20 (C-5’), 122.00 (C-6’).

**Sugar:** 101.20 (C-1’’), 71.60 (C-2’’), 74.40 (C-3’’), 70.02 (C-4’’), 77.70 (C-5’’), 61.50 (C-6’’).

**Quercetin (7)**

^**1**^**H NMR (400 MHz, DMSO-d6) δ (ppm): **7.68 (1H, d, J = 2.5 Hz, H-2'), 7.55 (1H, dd, J = 2.5 & 8.5, H- 6'), 6.92 (1H, d, J = 8.5 Hz, H-5'), 6.41 (d, J = 2 Hz, H-8), 6.19 (1H, d, J = 2 Hz, H-6).

^**13**^**C NMR (100 MHz, DMSO-d6) δ (ppm):** 176.29 (C-4), 164.33 (C-7), 161.18 (C-5), 158.99 (C-9), 156.58 (C-2), 148.15 (C-4′),145.51 (C-3′), 136.49 (C-3), 122.41, (C-1′), 120.49 (C-6′), 116.18 (C-5′), 115.44 (C-2′), 103.46 (C-10), 98.63 (C-6), 93.80 (C-8).

### Preparation of rubber composites

At room temperature, rubber and its constituents were combined in an open two-roll mill measuring 470 mm in diameter and 300 mm in operation distance. The slow roller’s gear ratio is 1:1.4, and its speed is 24 rpm. The rubber mixtures were allowed to vulcanize overnight. In an electrically heated press, rubber compounds were vulcanized at 142 ± 1 °C for NR and 152 ± 1 °C for SBR at a pressure of roughly 4 Mpa to produce vulcanized rubber sheets that were 2 mm thick. The sheets’ vulcanization time matches the ideal cure time Tc_90_ that was determined using the data from the curing curves.

### Rheometric characteristics

The vulcanization process was carried out, and the molds were made inaccordance with the unique cure times (Tc_90_) ascertained by use of TA Instruments’ MDR one (Moving Die Rheometer); USA at 142 ± 1 °C (NR) and 152 ± 1 °C (SBR) for 30 min. The curing characteristics obtained from the rheometric graph were minimum torque (M_L_), maximum torque (M_H_), torque extent (ΔM = M_H_-M_L_), scorch time (Ts_2_), and optimum cure time (Tc_90_). From determining the values ofTc_90_ and Ts_2_, the cure rate index (CRI) was calculated as follows:

1 $${\mathrm{CRI}} = {1}00/{\mathrm{Tc}}_{{{9}0}} - {\mathrm{Ts}}_{{2}}$$

### Mechanical properties of rubber composites

An electronic Zwick tensile testing machine (model Z010) from Germany was used to evaluate the mechanical properties of the manufactured NR and SBR composites in compliance with ASTM D412. Five samples are utilized and averaged to determine the mechanical parameters in terms of modulus at 100% elongation, elongation at break (%), and tensile strength (MPa). A Bareiss Shore A durometer (Germany) was used to measure the shore hardness of the produced composites in accordance with ASTM D2240 standard. Every measurement is made at room temperature, which is 25 ± 1 °C.

### Thermo-oxidative agingof rubber composites

In compliance with ASTM D 572-04, samples from rubber composites were subjected to a thermo-oxidative aging process by being placed in an air-circulating oven set at 90 ± 1 °C for two, four, six, and seven days. Tensile tests were conducted after the specimens had been stored at 25 °C for 24 h. Five samples were used to test mechanical qualities, and the average was then calculated.

### Swelling and crosslink density

The prepared rubber composites were left to swell in toluene for a full day in compliance with ASTM D 573-2007. Using the Flory-Rehner relation^42–47^, the molecular weight between crosslinks (Mc) was calculated using the following relation:

2 $$= \frac{1}{{\left( {2{\mathrm{Mc}}} \right)}} = \frac{ - 1}{{2_{\rho } V_{R} }} \times \left[ {\frac{{\ln \left( {1 - V_{R} } \right) + V_{R} + \mu V_{{R^{2} }} }}{{\left( {V_{{R^{1/3 - 1/2} }} V_{R} } \right)}}} \right]$$

NR density (ρ) is equal to 0.92 g/cm^3^ and NR-toluene interaction parameter (µ) is equal to 0.393. SBR density (ρ) is equal to 0.945 g/cm^3^ and SBR-toluene interaction parameter (µ) is equal to 0.446.

The crosslink density isdetermined according to the equation:

3 $$\nu = {1}/\left( {{\mathrm{2Mc}}} \right)$$

### Fourier transformsinfrared spectroscopy

The instrument used to record the FTIR was a Jascow FTIR-430 from Japan. Attenuated Total Reflection (ATR) method was used to measure FTIR of theprepared composites. The spectrum range of 4000 to 400 cm^−1^ was used to measure the data.

### Field emission scanning electron microscope

Surface of the prepared NR and SBR composites were takenby SEM, Quanta FEG-250.

## Results and discussion

### Characterization of the extract of *Acacia nilotica* leaves

#### Fourier transforms infrared spectroscopy

**Fig. 1:**
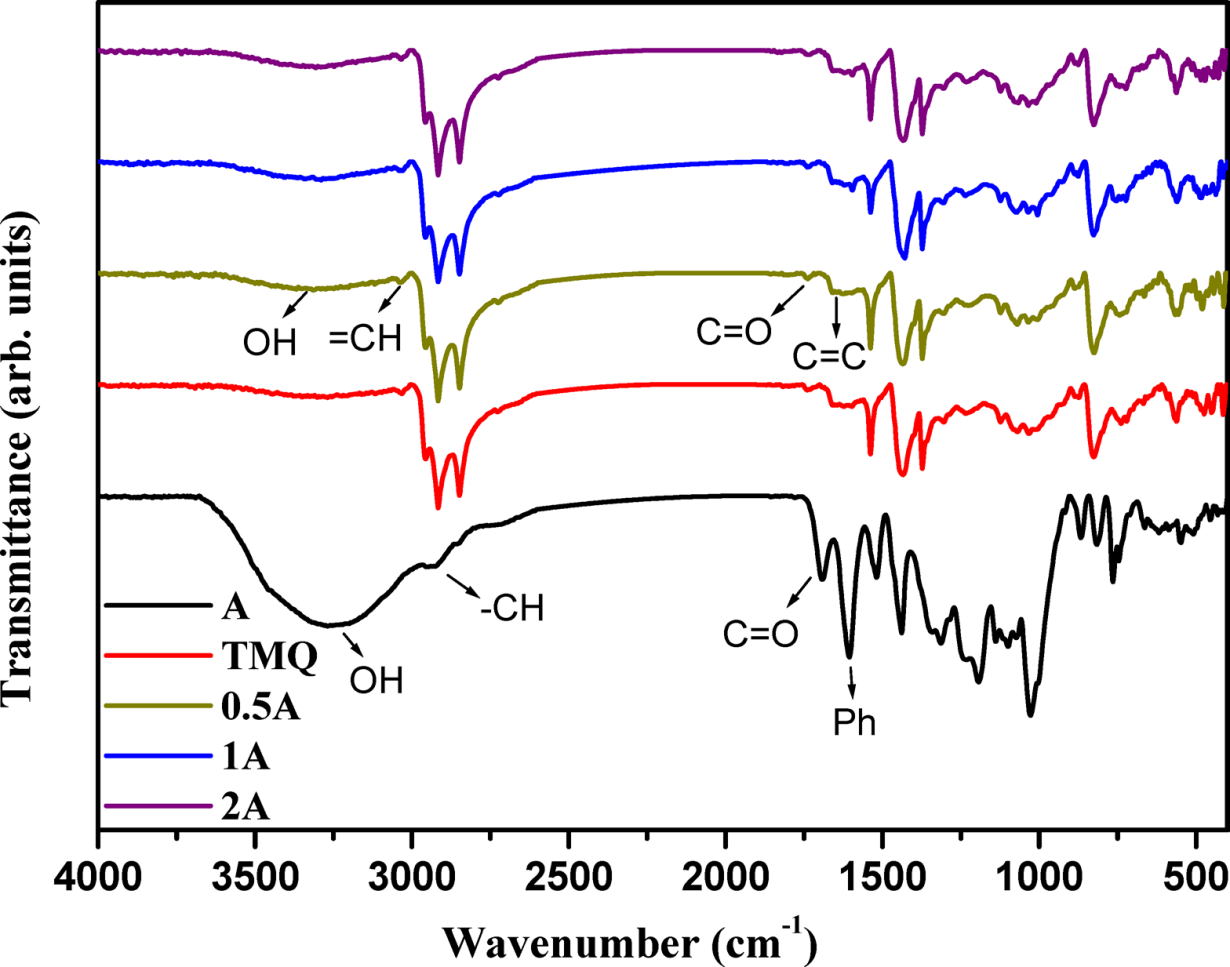
FTIR of *A. nilotica* leaves extract (A) and NR composites.

FTIR of the extract of *A. nilotica* leaves reveals the presence of hydroxyl group at the peak 3252 cm^−1^. The aromatic = CH group appears at 3051 cm^−1^. The peaks at 2936 cm^−1^ and 2857 cm^−1^ are attributed to the aliphatic –CH group. The carbonyl group appears at 1695 cm^−1^. The peak at 1609 cm^−1^ represents the phenyl group, as shown in Fig. 1. The presence of all these peaks proves the structure of the extract.

#### Field emission scanning electron microscope

**Fig. 2:**
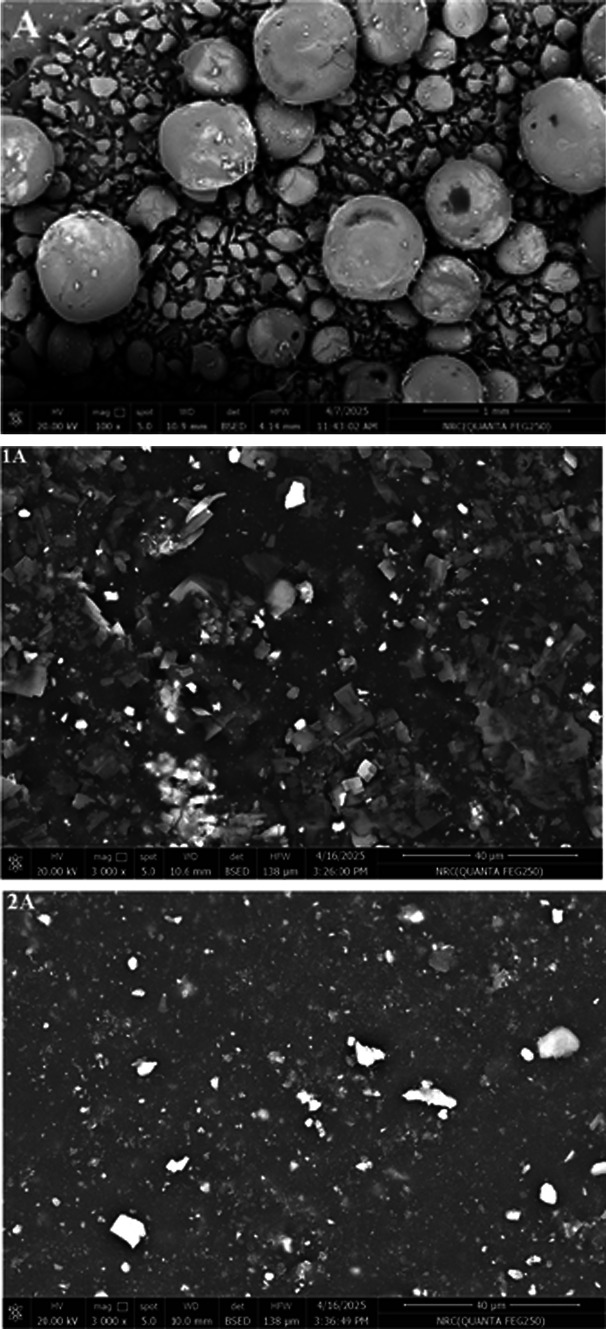

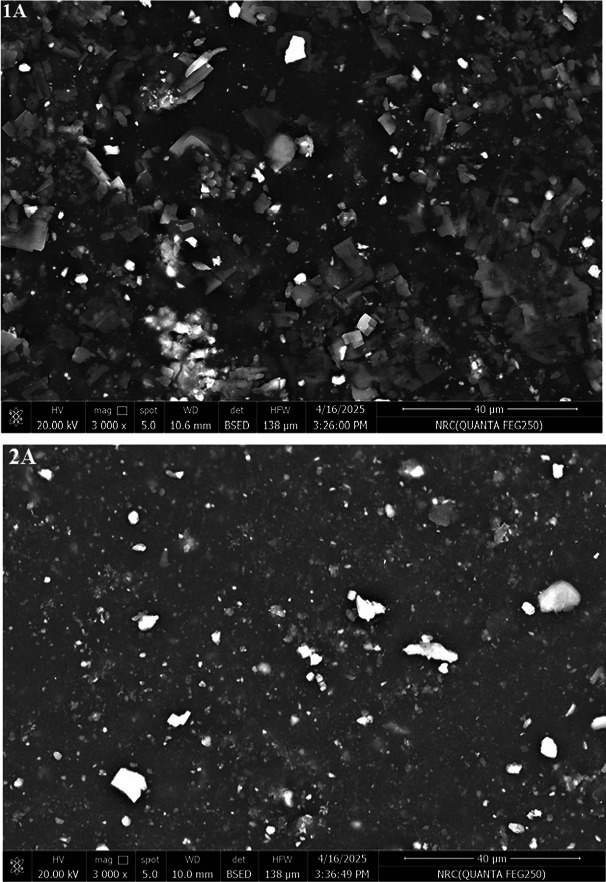
FESEM of *A. nilotica* leaves extract (A) and NR composites.

The surface image of the material is represented in Fig. 2. It reveals the presence of spherical particles and stones-like particles.

### Characterization of rubbercomposites

The extract of *A. nilotica* leaves with different concentrations (0.5–2 phr) compared to 1 phr TMQ as the commercial antioxidant was incorporated in the standard formula of NR and SBR (Table 1). The rubber composites are characterized by FTIR and FESEM. The rheometric characteristics, as well as the physico-mechanical properties, of thecomposites before and after thermo- oxidativeaging at 90 °C for different aging times (1–7 days) are measured.

**Table 1:** Rubber formulations.

2A	1.5A	1A	0.5A	TMQ	Rubber	Sample/ingredient, phr^a^
100	100	100	100	100	100	Rubber^b,c^
–	–	–	–	1	–	TMQ
2	1.5	1	0.5	–	–	A^d^

^a^Part of hundred parts of rubber; ^b^NR or SBR; ^c^Base recipe: ZnO 5, Stearic acid 2, HAF 20, Processing oil 3, CBS 1.5, S 2; ^d^*Acacia nilotica* leaves extract.

#### Effect of *Acacia nilotica* leaves (A) extract on the properties of natural rubber (NR) composites

##### Fourier transforms infrared spectroscopy

FTIR of NR composites containing *A. nilotica* leaves extract compared to TMQ reveals the presence of the A imbedded in the structure of rubber, as illustrated in Fig. 1. The peak at 3309 cm^−1^ is corresponding to the phenolic OH groups in *A. nilotica* leaves resulted in the isolation of gallic acid 1, ethyl gallate 2, catechin 3, quercetin 3-O-rhanmnopyranosyl (1–6) glucopyranoside 4, quercetin-3-O-α-L-rhamnopyranoside 5, quercetin 3-O-β-glucopyranoside (6), and quercetin 7 (S1). The band at 3036 cm^−1^ is attributed to = CH group. The peaks at 2921 cm^−1^, and 2843 cm^−1^ are due to the vibrations of aliphatic –CH group. The vibration of the carbonyl group of *A. nilotica* leaves extract appears at 1738 cm^−1^. The peak at 1638 cm^−1^ reveals the presence of C=C group of NR. The peak at 1607 cm^−1^ is due to the phenyl ring of the A. The presence of the functional groups of the A besides that of NR proves the structure of the composites.

##### Field emission scanning electron microscope of the NR composites

The surface images of NR containing A. extract are shown in Fig. 2. NR composite containing TMQ shows relatively large filler agglomerates. This may be attributed to poor filler dispersion or rubber–carbon black interfacial bonding. The incorporation of 0.5A in NR/carbon black shows a finer and more homogeneous distribution of carbon black particles compared to TMQ, indicating better filler–rubber compatibility. On the other hand, the increasing concentration of A leads to large agglomerations.

### Rheometric characteristics

The results of Rheometric characteristics such as M_L_, M_H_, Ts_2_, Tc_90_, CRI andthe value of cure extent, which is related to the crosslinked density of the composites, were calculated as difference between maximum and minimum torque values of the cure curves (M_H_-M_L_) are measured and listed in Table 2. It can be seen from Table 2 that the sample containing 1phr TMQ has the lowest value for M_L_, M_H_, and M_H_-M_L_ compared to control (NR). The samples containingthe extract of *A. nilotica* leaves(0.5-2phr) show slightly reduced M_L_ values, indicating a minor decrease in compound viscosity. This suggests that the extract of *Acacia nilotica* leaves may provide some plasticizing effect at higher concentrations. The samples containing Acacia extracted (0.5–2phr) show higher M_H_ compared to NRand TMQ samples, the highest value observed for sample contains 1.5phr of A (11.84 N/mm^2^). This indicates improved crosslink density and stronger network formation when the extract of *A. nilotica* leaves (1.5phr) is incorporated to natural rubber. The crosslinking efficiency was measured by the torque difference (M_H_-M_L_), one can be seen from Table 2 that the difference increases with incorporation of A. This trend supports the reinforcing effect of the extract of *Acacia nilotica* leaves, due to its phenolic compounds acting as antioxidants or promoting crosslinking^34^.

**Table 2:** Rheometric characteristics and swelling properties of the prepared rubber samples at room temperature of NR compounds containing *Acacia nilotica* leaves extract with different concentrations.

Sample No./property	NR	TMQ	A0.5	A1	A1.5	A2
M_L,_ N/mm^2^	0.2	0.11	0.19	0.15	0.15	0.14
M_H_, N/mm^2^	11.05	9.80	11.15	11.41	11.84	11.48
M_H_-M_L_	10.85	9.69	10.96	11.26	11.69	11.34
Ts_2_, min	4.39	5.06	4.84	4.70	4.66	4.82
Tc_90_, min	12.22	12.94	12.63	10.25	11.76	11.92
CRI, min^−1^	12.77	12.69	12.84	18.02	14.08	14.08
Swelling properties						
Qm,	3.14	3.33	3.13	3.10	3.06	3.21
Mc, (g/mol)	4061	4523	4037	4001	3873	4228
*v*, mol/cm^3^ × 10^–6^	12.3	11.1	12.4	12.5	12.9	11.8

The scorch time remained as the same for all the samples, whereasthe optimum cure time Tc_90_ and cure rate index (CRI) werenot much variation for samples (NR/HAF, NR/HAF/TMQ, andNR/HAF/0.5A). On the other hand, sample 1A had the lowest Tc_90_ and the highest CRI. Also, one can see the samples1.5A, 1A and 2A had the lowest and the highest Tc_90_ and CRI, respectively. It can be concluded that the extract of *A. nilotica* leaves at concentration 1 phr (1A) may be used as a secondary accelerator for natural rubber. It is noted that the CRI increases from 12.77 (NR) to 18.02 (1A), then decreases slightly at higher concentrations. This suggests that low concentrations of the extract enhance curing kinetics, while higher concentrations may hinder the reaction due to excessive antioxidant activity or steric effects.

### Swelling properties

The swelling study was carried out to show the impact of the extract of *A. nilotica* leaves on the swelling properties and crosslink density. From Table 2, it can be seen that the equilibrium swelling and the molecular weight between crosslinks (Mc) values for the 0.5A, 1A, 1.5A and 2A compositesrange from (3.13–3.41) and (4037–4228), respectively but NR vulcanizate (3.14,4061) and TMQ vulcanizate (3.33, 4523). On the other hand, the crosslink density values increased with incorporation of the extract of *A. nilotica* leaves but decreased with TMQ compared to the control sample. The highest value (12.9 × 10⁻⁶ mol/cm^3^) was observed for sample 1.5A, indicating the densest network and strongest crosslinking at this concentration. The lower crosslink density value can be attributed to the presence of certain impurity molecules from the extract of *Acacia nilotica* leaves which obstructs the formation of crosslinking.

### Physico-mechanical properties

**Fig. 3:**
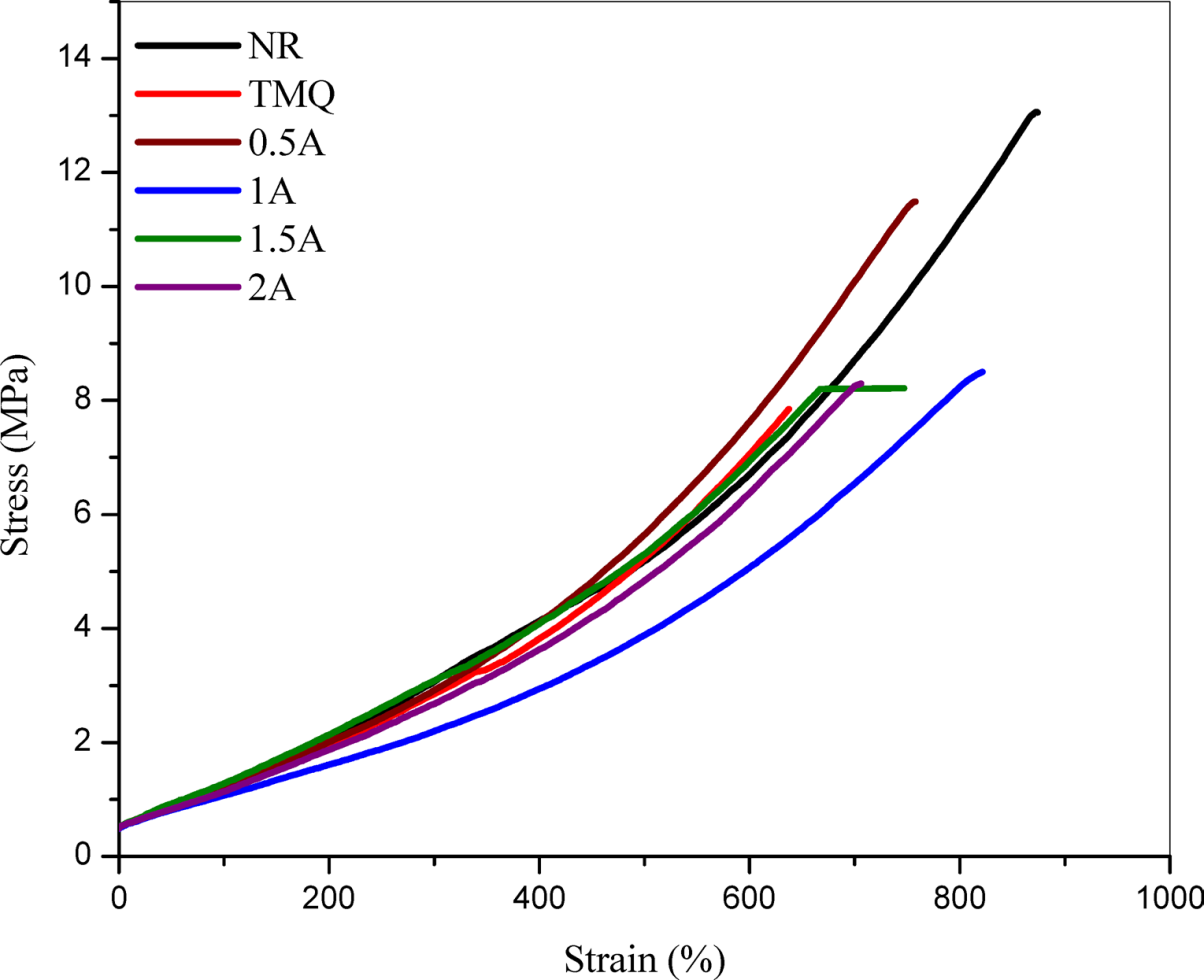
Stress–strain of NR composites.

The effect of incorporating the extract of *A. nilotica* leaves extract at different concentrations (0.5, 1, 1.5, and 2 phr) on the physico-mechanical properties of NR compositesare shown instress-strain curves represented in Fig. 3. The data is also summarized in Table 3, and compared with the control sample (NR without antioxidant) and with a conventional antioxidant (TMQ). According to Table 3, it is clearly shown that the tensile strength of NR vulcanizate (control) is higher than the composites containingthe extract of *A. nilotica* leaves with different concentrations (0.5–2 phr), and TMQ has the lowest value. The elongation at break for NR compositescontaining A (the extract of *A. nilotica* leaves with different concentrations 0.5-2 phr) has the same trend of tensile strength. The values of modulus at different elongation (50 & 100) of the NR composites with and without TMQ and the extract of *A. nilotica* leaves are similar values but modulus at 300 elongations is slight decrease. On the other hand, the values of hardness increased steadily with the extract of *A. nilotica* leaves concentration, from 58 (0.5A) to 68 (2A), suggesting enhanced stiffness at higher loadings. TMQ sample produced the softest composites.

**Table 3:** Physico-mechanical properties of NR vulcanizates containing the extract of *Acacia nilotica* leaves with different concentrations.

Sample No./property	NR	TMQ	A0.5	A1	A1.5	A2
TS, MPa	13.06	7.84	11.49	8.5	8.2	8.29
Elongation at break, %	822	618	757	752	747	706
Modulus at 50 elongation, MPa	0.84	0.85	0.83	0.81	0.87	0.84
Modulus at 100 elongation, MPa	1.18	1.20	1.13	1.11	1.22	1.16
Modulus at 300 elongation, MPa	3.07	2.85	2.92	2.35	2.79	2.65
Hardness, Shore A	61	57	58	60	65	68

### Influence of thermo-oxidative aging on the mechanical properties

Rubber and rubber networks degrade as a result of the energy that is provided in the form of heat, light, oxygen, and ozone. Free radicals start the oxidation processes, which are then stopped by recombination or by utilizing stabilizers to render the radicals inactive. Determining the technically significant features of elastomers is crucial since, in theory, the aging of polymer networks is thought to be related to or explainable by changes in the network structure^4,6,11,12^.

**Fig. 4:**
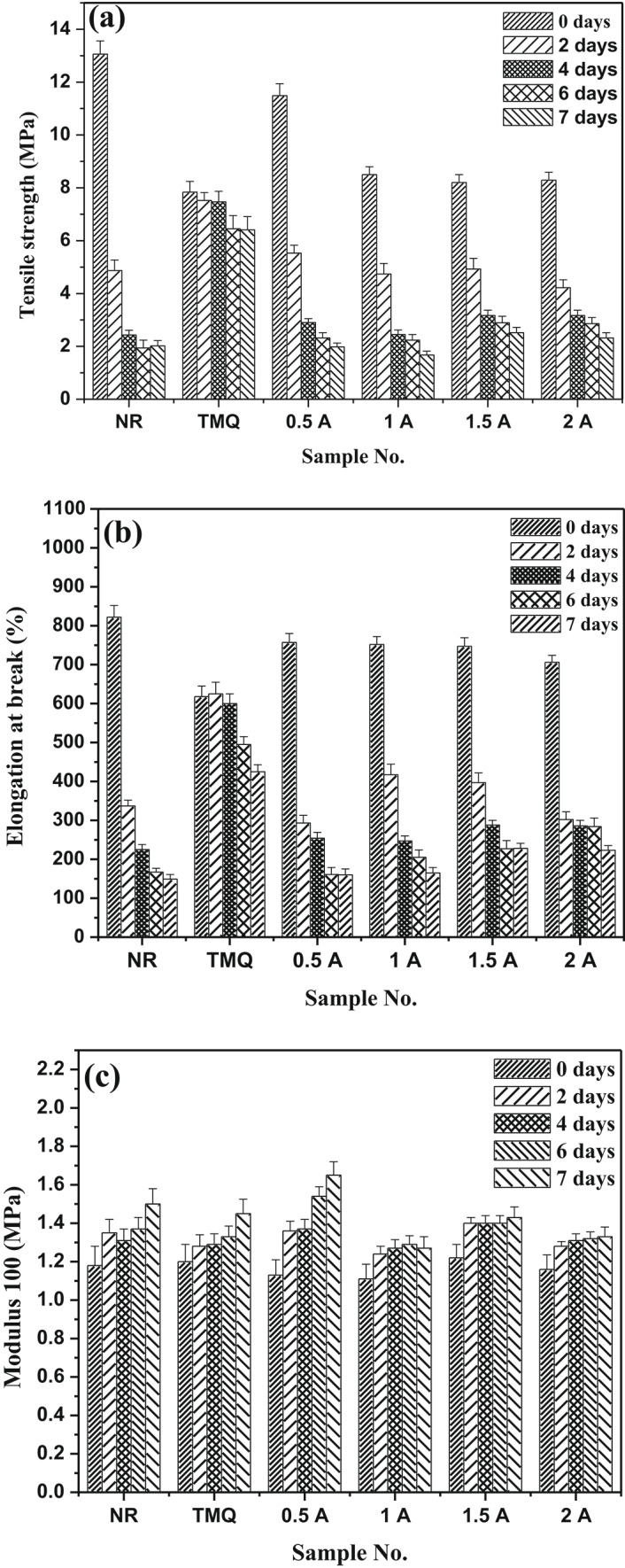
(**a**–**c**) Mechanical properties after aging of NR composites; (**a**) Tensile strength; (**b**) Elongation; (**c**) Modulus.

The natural rubber mixes containing the TMQ and the extract of *A. nilotica* leaves (A) were vulcanized at 142 ± 1 °C up to their optimum cure time and subjected to accelerated thermo-oxidative aging in an oven at 90 ± 1 ⁰C for different periods of time up to7 days. One can be seen from Fig. 4a that NR composites (control) show rapid decrease butTMQ sample provides better stability than NR but starts at lower baseline strength. Addition of 0.5 phr of A to NR/HAF keeps tensile strength better than NR/HAF and NR/HAF/TMQ composite over agingtime; degradation rate is slower. As the concentration of the extract of *A. nilotica* leaves increased (1–2 phr), the tensile strength begins to decrease and continue to drop with aging time (not as protective as 0.5A). Also, it is noticed that after 7 days, NR drops to the lowest strength, TMQ sample retains stable but composites 0.5A phr remains the best performer, retaining higher tensile strength than both NR and TMQ. Figure 4b shows the influence of thermo-oxidative aging (2-7 days) on the elongation at breakof NR composites containing the extract of *A. nilotica* leaves with different concentrations and commercial antioxidant (TMQ). It can be seen that, NR vulcanizate (with antioxidant) shows rapid decrease in elongation, TMQ vulcanizatehas better retention, 0.5A compositesretains elongation far better than NR and better than TMQ, while retention is reasonable, a higher concentration causes a quicker drop than 0.5A. In comparison to 0.5A, retention is accepted, but a high concentration of the extract of *A. nilotica* leaves causes a faster drop. Figure 4c shows the changes in the modulus at 100% elongation with aging time (2–7 days) at 90 °C for NR composites. One can see from the figure that all thecomposites show an increase in modulus with increasing aging time due to formation of extra crosslinking, hardening, and increasing stiffness. Vulcanizate contains 0.5 phr of the extract of *A. nilotica* leaves has the best stability. Higher concentrations (1.5A–2A) tend to stiffen but still offer protection.

### Effect of the extract of *Acacia nilotica* leaves (A) on the properties of styrene butadiene rubber (SBR) composites

#### Fourier transforminfrared spectroscopy of SBR composites

**Fig. 5:**
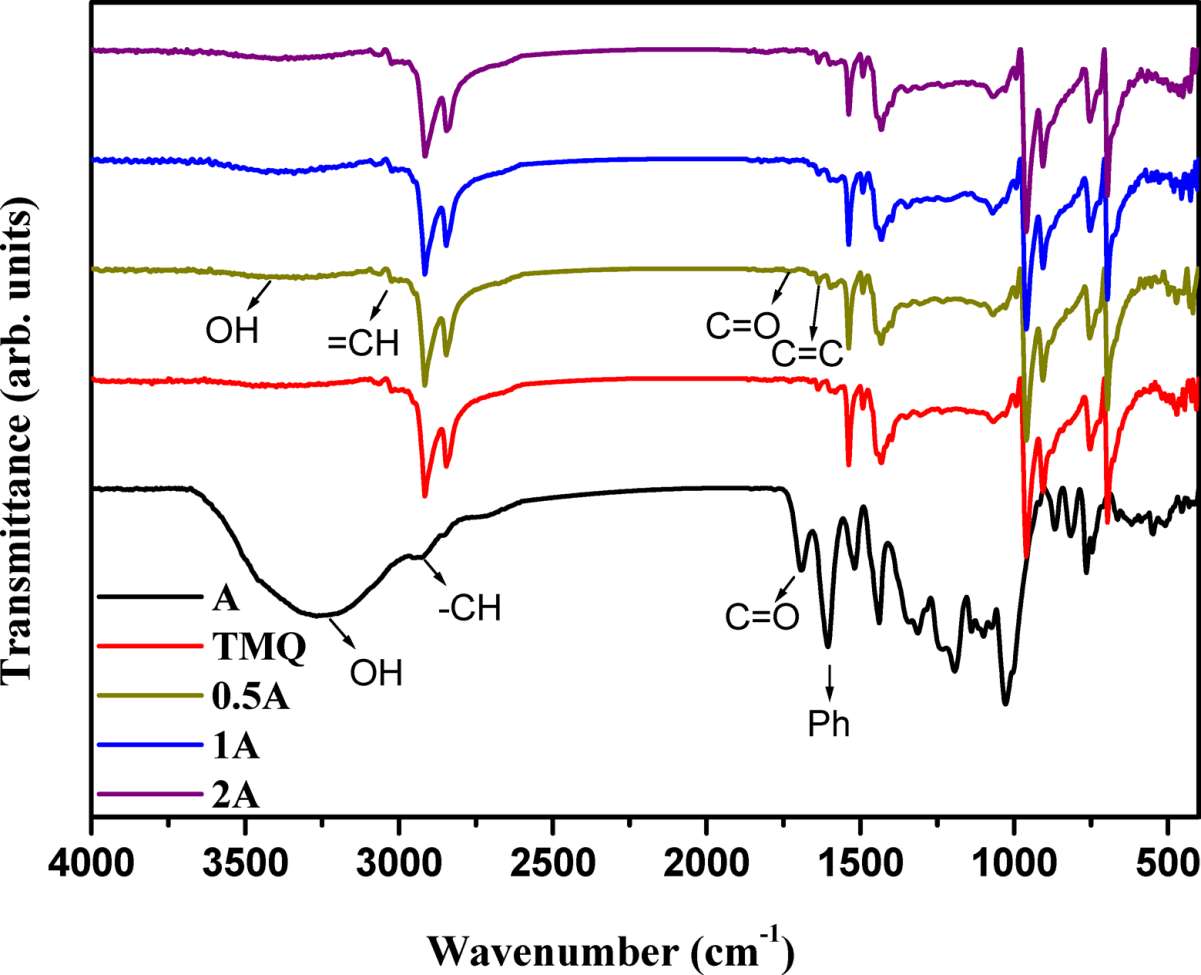
FTIR of *Acacia nilotica* leaves extract (A) and SBR composites.

Figure 5 reveals the presence of the A imbedded in the structure of rubber for the composites 0.5A, 1A, and 2A. The peak at 3416 cm^−1^ is corresponding to the hydroxyl group of phenolic compounds present in the extract of *A. nilotica* leaves (not present in TMQ). The band at 3015 cm^−1^ is attributed to = CH group. The peaks at 2929 cm^−1^, and 2843 cm^−1^ are due to the vibrations of aliphatic -CH group. The vibration of the carbonyl group of the A appears at 1717 cm^−1^. The peak at 1638 cm^−1^ reveals the presence of C = C group of SBR. The peak at 1595 cm^−1^ is due to the phenyl ring of SBR. The presence of the functional groups of the A besides that of SBR proves the structure of the composites.

#### Field emission scanning electron microscope

**Fig. 6:**
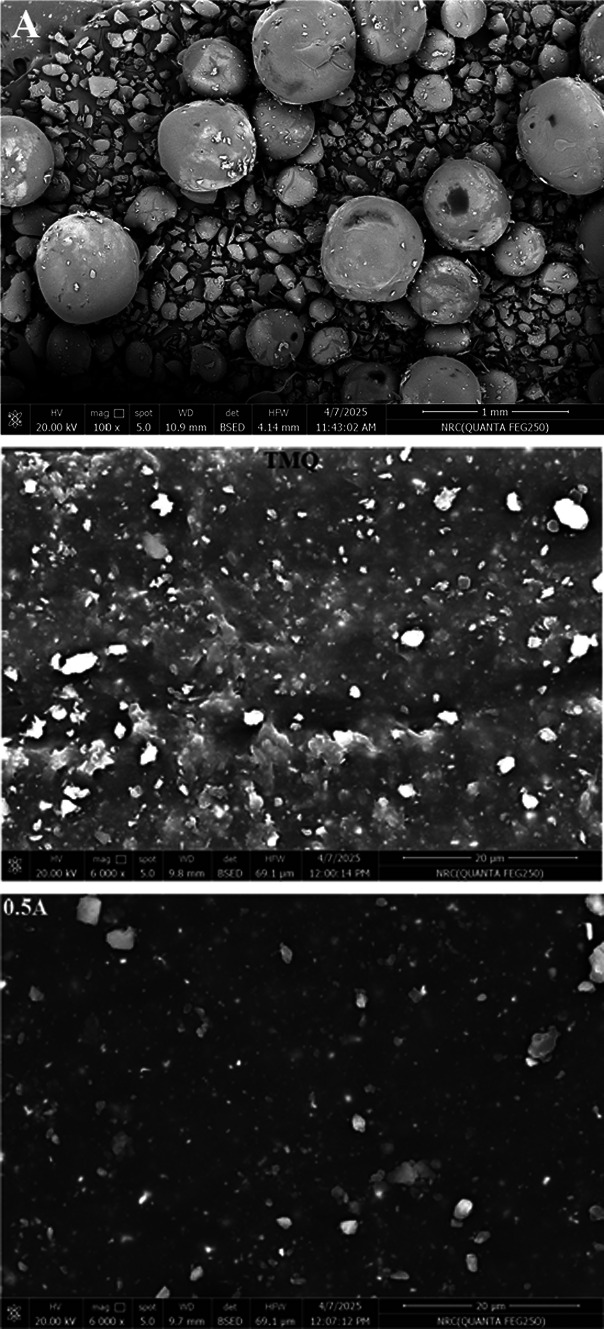

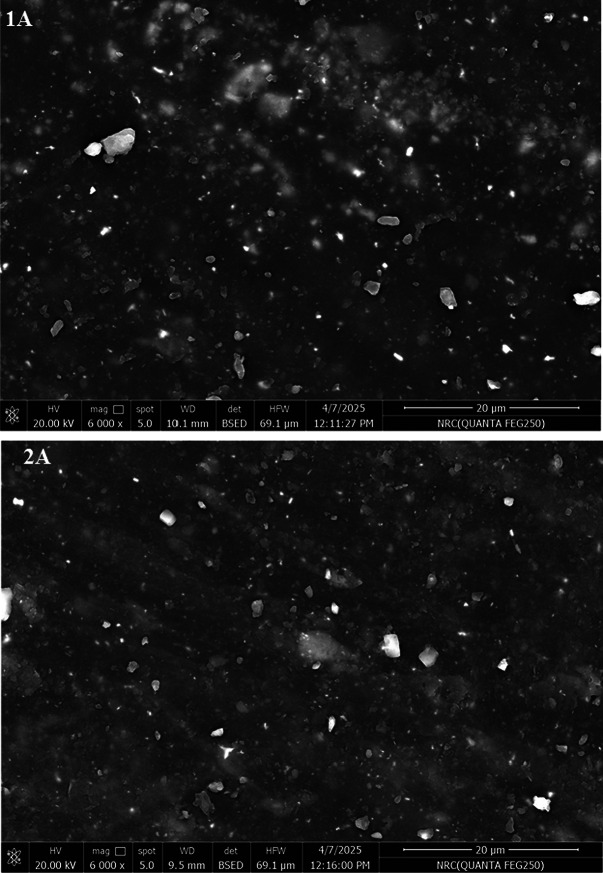
FESEM of *Acacia nilotica* leaves extract (A) and SBR composites.

Figure 6 shows the surface images of SBRcomposites containing the extract of *A. nilotica* leaves (A). It notices that the surface of the vulcanizate containing the commercial antioxidant (TMQ) is quite rough, with big agglomerates and uneven particle dispersion. On the other hand, the addition of A in SBR/carbon black improved particle dispersion, 1A and 2A composites, show smoother, more uniform morphology compared to TMQ. This may be attributed to phenolic OH groups present in the extract of *A. nilotica* leaves (A) can form hydrogen bonding or π–π interactions with carbon black surfaces and styrene domains in SBR.

### Cure characteristics

The effect of *A. nilotica* leaves extract (A)with different concentrations (0.5–2 phr)compared to 1 phr TMQ on the parameters of curing such as minimum(M_L_), maximum (M_H_), difference torque(∆M), scorch time (T_S2_), optimum cure time(T_C90_), and cure rate index(CRI) of the carbon-filled SBR are listed in Table 4. One canobserve that there is not much variation inminimum torque of SBR/HAF compounds withdifferent concentrations of *A. nilotica* leaves extract comparing with the SBR and TMQ samples i.e. nearly similar in processability. On the other hand, a gradual reduction in the values of maximumtorque, difference torque is observed with increasing concentrations of *A. nilotica* leaves extract. The cure time (Tc_90_) and scorch time (Ts_2_) decline marginally with increasing extract content, but they both stay within industrially acceptable ranges.

**Table 4:** Rheometric characteristics and swelling properties of the prepared rubber samples at room temperature of SBR compounds containing the extract of *A. nilotica* leaves with different concentrations.

Sample No./property	SBR	TMQ	A0.5	A1	A1.5	A2
M_L,_ N/mm^2^	0.97	1.01	1.01	0.97	1.02	0.96
M_H_, N/mm^2^	11.50	12.47	11.96	10.16	10.12	10.01
M_H_-M_L_	10.53	11.46	10.91	9.64	9.10	9.05
Ts_2_, min	9.78	9.34	9.38	9.16	8.27	8.79
Tc_90_, min	17.28	16.79	17.18	17.27	16.82	17.39
CRI, min^−1^	13.33	13.423	12.82	12.33	11.69	11.63
Swelling properties						
Qm,	2.86	2.93	2.97	3.04	3.12	3.22
Mc, (g/mol)	4705	4939	5070	5310	5591	5953
*v*, mol/cm^3^ × 10^–5^	10.6	10.1	9.86	9.42	8.94	8.4

With higher A content, the cure rate index (CRI) exhibits a little decrease, suggesting a slower rate of curing. A reduction in curing properties may be attributed to the interference of sulfur-based vulcanization chemistry with natural compounds (such as tannins or polyphenols) from *A. nilotica*, which may scavenge free radicals or change accelerator action.

### Swelling properties

The swelling properties and crosslink density values of the SBRcompositescontaining with and without the extract from *A. nilotica* leaves with different concentrations(0.5-2phr) andcommercial antioxidant TMQ were evaluated and tabulated in Table4. It isobvious that, the SBR/HAFcomposite realizes lower swelling percentage than the composites contain TMQ and the extract from *Acacia nilotica* leaves. Fundamentally, crosslink density refers to the degree of crosslinking which determines the state of cure of a compound. Also from this table, it can be seen that the crosslink density value for SBR/HAF/TMQ composite shows similar value to that of control but SBR/HAF/A (0.5-1 phr) composites have slightly lower crosslink density than SBR/HAF. On the other hand, the values of crosslinking density decreased as the concentration of the extract from *A. nilotica* leaves increased. The diminished degree of crosslinking can be explained by the presence of specific contaminant molecules derived during leaf extraction^34^. Consequently, creating crosslinks across these chains becomes a challenging task.

### Physico-mechanical properties of styrene butadiene rubber composites

**Fig. 7:**
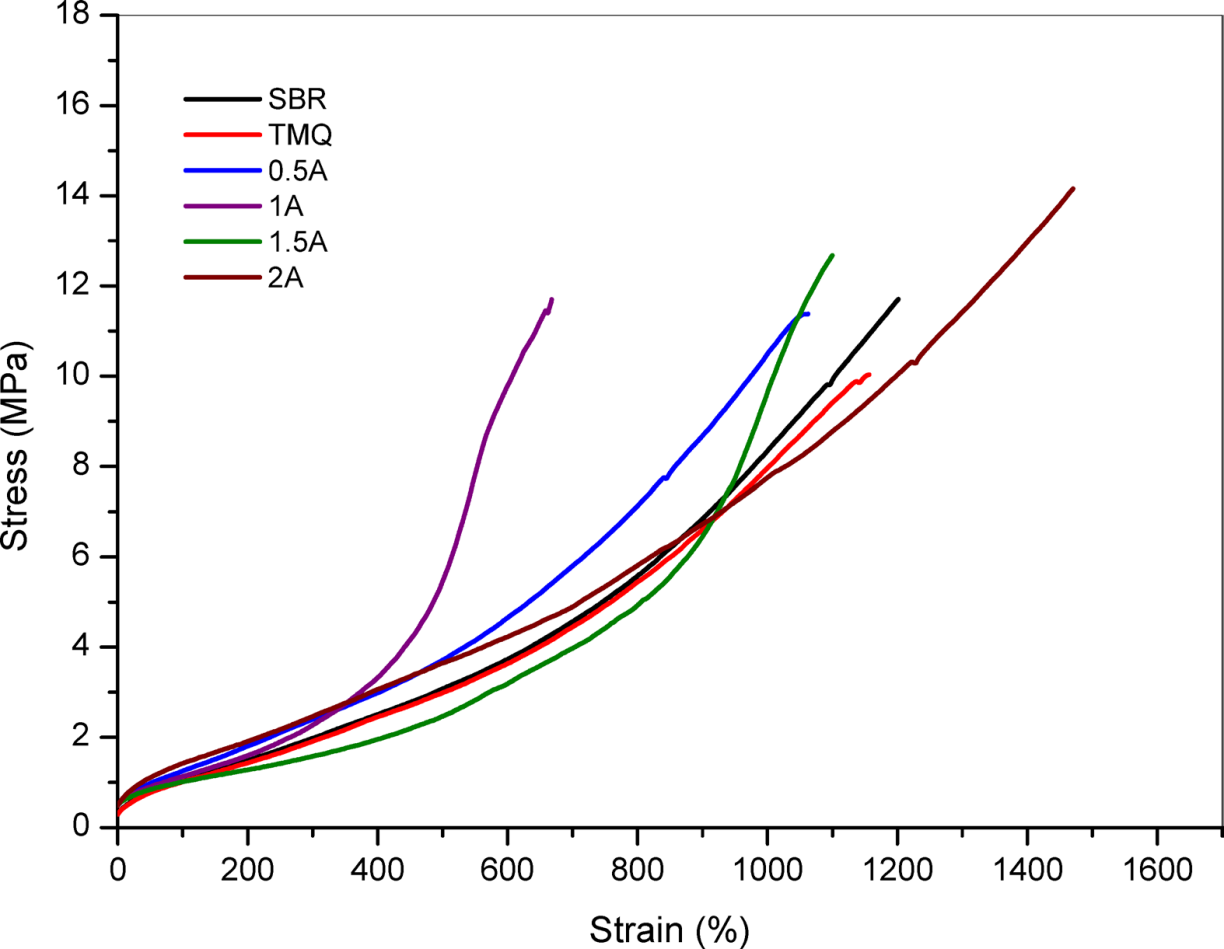
Stress–strain of SBR composites.

Physico-mechanical properties as represented by tensile strength, elongation at break and modulus at 100%, 300%, 500% elongation (M50, M100, M300) for each SBR/HAF composites were measured and shown in Table 5. The stress–strain curves for the prepared SBR/HAF rubber composites with TMQ and the extract from *A. nilotica* leaves (A) are shown in Fig. 7. SBR/HAF/TMQ composite had lower tensile strength than neat rubber composite but elongation at break and modulus did not be affected. On the other hand, the incorporation of the extract of *A. nilotica* leaves in SBR/HAF mixes with 0.5 and 1 phr concentration have the same value of SBR/HAF composites. As the concentration of A increased the value of tensile strength increased. The elongation at break value of the SBR/HAF, SBR/HAF/TMQ and SBR/HAF/A (0.5–1.5 phr) composites is slightly improved but increased for SBR/HAF/A (2phr).

**Table 5:** Physico-mechanical properties of SBR vulcanizates containing the extract of *A. nilotica* leaves with different concentrations.

Sample No./Property	SBR	TMQ	A0.5	A1	A1.5	A2
TS, MPa	11.7	10.03	11.38	11.69	12.66	14.15
Elongation at break, %	1016	1034	1027	1000	1069	1066
Modulus at 50 elongation, MPa	0.8	0.78	0.93	0.93	0.95	1.11
Modulus at 100 elongation, MPa	1.06	1.02	1.16	1.22	1.23	1.31
Modulus at 300 elongation, MPa	1.99	1.93	2.15	2.28	2.29	2.33
Hardness, Shore A	60	60	63	65	62	62

The value of tensile modulus (M50, M100, and M300) of SBR/HAF composites with different concentration from the extract of *A. nilotica* leaves has higher values than SBR/HAF and SBR/HAF/TMQ, indicating better stiffness and reinforcement. By the addition of the extract of *A. nilotica* leaves, the hardness value of SBR/HAF composites increased. It is noted that the addition of the extract of *A. nilotica* leaves, especially at higher concentrations (1.5A–2A), shows superior reinforcement and antioxidant efficiency compared to the commercial TMQ in SBR composites. The values of hardness for all composites are similar.

### Physico-mechanical properties of SBR composites after thermo-oxidative aging at 90 °C for different time periods (2–7 days)

**Fig. 8:**
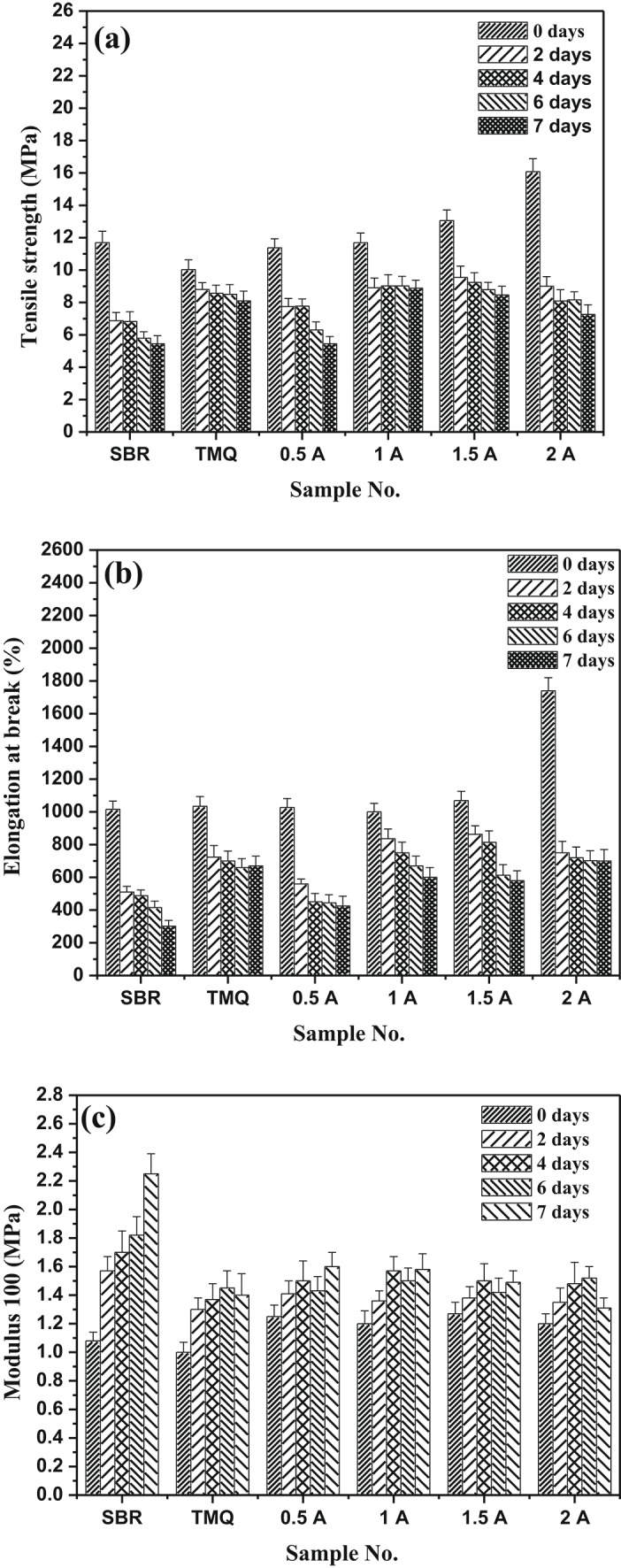
(**a**–**c**) Mechanical properties after aging of SBR composites; (**a**) Tensile strength; (**b**) Elongation; (**c**) Modulus.

Figure 8a–c illustrates the physico-mechanical properties of SBR/HAF, SBR/HAF/TMQ and SBR/HAF/A(0.5-2 phr) composites after thermo-oxidative aging process. One can see reduction in both tensile strength, elongation at break values but the values of modulus (100%) increased after aging for all the SBR/HAF rubber composites. SBR/HAF composites had sharp decrease in tensile strength but SBR/HAF composite with TMQ slows the decline with increasing aging time. On the other hand, the SBR/HAF/A composites have better retention of tensile strength than both SBR and TMQ, especially at0.5A–1A phr. At higher concentrations (1.5–2 phr), the protection is still present but it is less effective, possibly due to agglomerations or plasticization effects. Compared with TMQ, the extract of *A. nilotica* leaves performs better in long-term stability and maintains higher initial tensile strength. These changes in the physico-mechanical properties indicate that rubber has undergone degradation. Under exposed rubber composites for temperature of 90 °C, the rubber chain cleaves due to the formation of new crosslinks such as mono-sulphidic and destroyed poly-sulphidic crosslinks. This mono-sulphidiccrosslinks have low resistance to heat orthermal aging. Meanwhile, the increasing in modulus value isattributed tothe stiffness of the rubber matrix due to the reduction in the number of double bonds^34^.

Figure 8b shows the dependence of elongation at break of rubber composites on the aging time. From this figure, it is obvious that SBR/HAF composite hassignificant oxidative stiffening and a sharp decrease in elongation but composite SBR/HAF/TMQ begins lower, and maintains elongation marginally better. On the other hand, the composites with 0.5 phr of the extract of *A. nilotica* leaves has best retention then composites with 1–2 phr of the extract of *A. nilotica* leaves. After seven days, the vulcanizate with 0.5 phr has best retention;composites with 1–2 phr of the extract of *A. nilotica* leaves are less effective but better than from SBR composite and composite with TMQ. The extract from *A. nilotica* offers robust protection, particularly at 0.5 phr, extending the flexibility of SBR. After thermo-oxidative aging (2–7 days), the values of modulus at 100% elongation increases with aging for all SBR/HAF/A composites (Fig. 8c).

Therefore, there exists a clear need for a comprehensive and comparative investigation that evaluates the structure–property–aging relationships of *A. nilotica* leaf extract in both NR and SBR composites. The present work addresses this gap by systematically examining curing characteristics, morphology, swelling behavior, and thermo-oxidative aging resistance across a wide concentration range, while directly comparing performance with a commercial antioxidant. This approach is essential to establish formulation guidelines, identify optimal loading levels, and assess the feasibility of *A. nilotica* extract as a sustainable and industrially relevant antioxidant for rubber applications.

## Conclusion

The extract of *A. nilotica* leaves improves cure properties and crosslinking efficiency when added to NR compounds, especially at 0.5–1.5 phr. However, the 0.5 phr of the extract of *A. nilotica* leaves provided the best balance of physico-mechanical properties. The tensile strength and elongation at break values were improved than TMQ. At greater concentrations (≥ 1 phr), hardness increased but tensile strength and elongation decreased, indicating stiffer but less extensible composites. This demonstrates that *A. nilotica* extract can act as a natural antioxidant in NR and 0.5 phr was the optimal concentration for maintaining optimum mechanical characteristics. On the other hand, the polyphenolic compounds present in the extract of *A. nilotica* leaves, make the SBR composites behave largely as a cure retarder and network diluent at the dosages0.5–2 phr. The physico-mechanical properties of SBR vulcanizates before and after aging were significantly influenced by the incorporation of the extract of *A. nilotica* leaves. The SBR without an antioxidant exhibited a comparatively high modulus, moderate tensile strength, and elongation at break. Tensile strength and elongation were enhanced by the addition of A. nilotica extract. The optimal performance was noted at 0.5–1 phr compared to the commercial antioxidant TMQ. Mechanical strength was slightly reduced with higher extract loadings (≥ 1.5 phr) which may be due to excessive filler–matrix interactions.

## Supplementary Information

Below is the link to the electronic supplementary material.

Supplementary Material 1

## Data Availability

The datasets used and/or analysed during the current study available from the corresponding author on reasonable request.
