# Supplementary material for: Insight on the impact of Acacia nilotica leaves extract on the characteristics of natural and synthetic rubber composites
Source: Sci Rep. 2026 Mar 19;16:9530. doi: 10.1038/s41598-026-40512-8 (PMC13004932; doi:10.1038/s41598-026-40512-8)
Supplement: Supplementary file 1 — Supplementary Material 1 [file 41598_2026_40512_MOESM1_ESM.docx]

Supplementary materials:

S1 Chemical structures of the isolated compounds from Acacia nilotica leaves extract.

S2 ^1^HNMR (a) and ^13^CNMR (b) of catechin (3).

S3^1^HNMR (a) and ^13^CNMR (b) of quercetin 3-O-α-L-rhamnopyranosyl-(1→6))-β-D-glucopyranoside (4).

S4 ^1^HNMR (a) and ^13^CNMR (b) ofquercetin-3-O-α-L-rhamnopyranoside (5).

S5 ^1^HNMR (a) and ^13^CNMR (b) of quercetin 3-O-β-D-glucopyranoside (6).

S6 ^1^HNMR (a) and ^13^CNMR (b) of quercetin (7).

S1


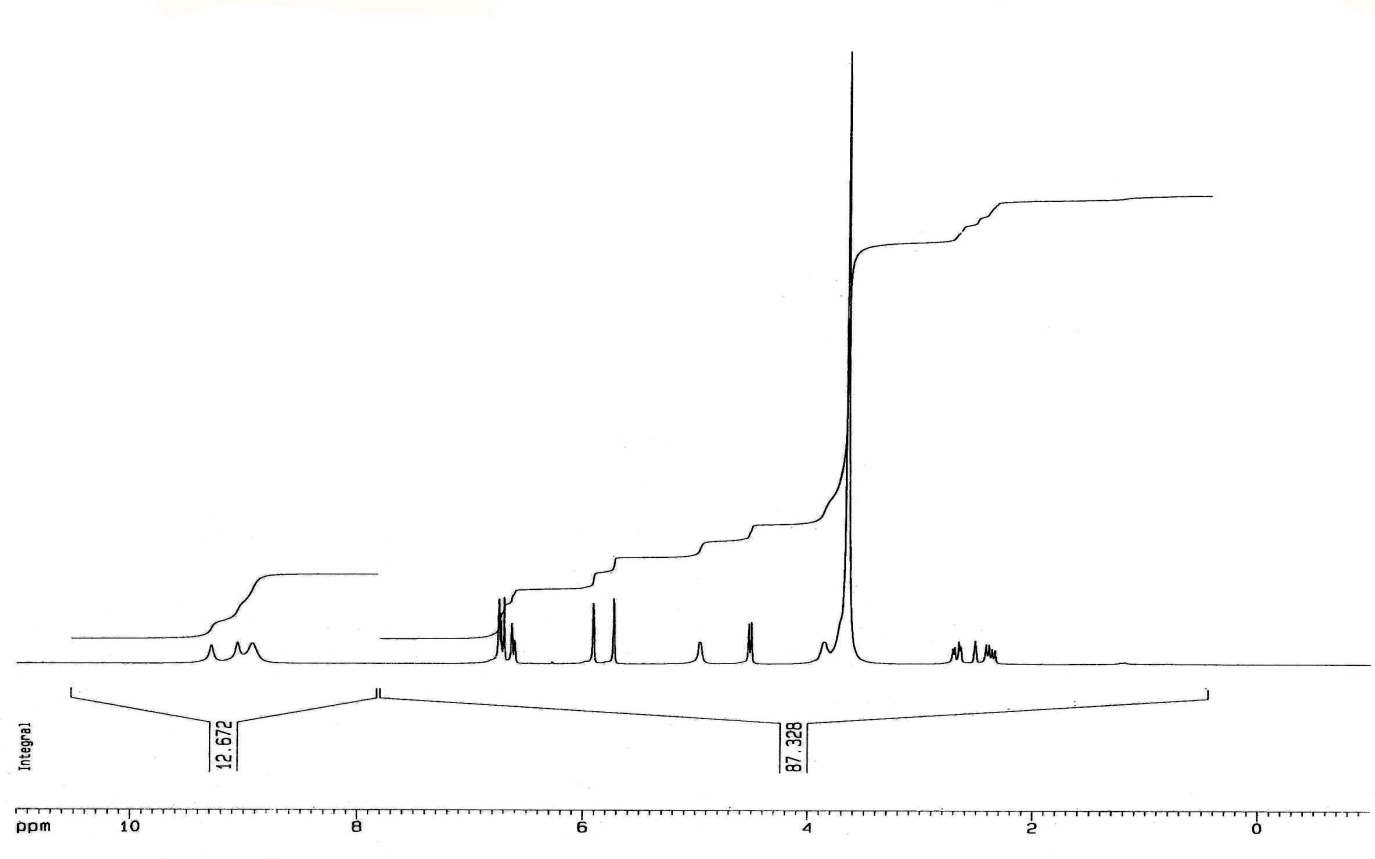


**(a)**


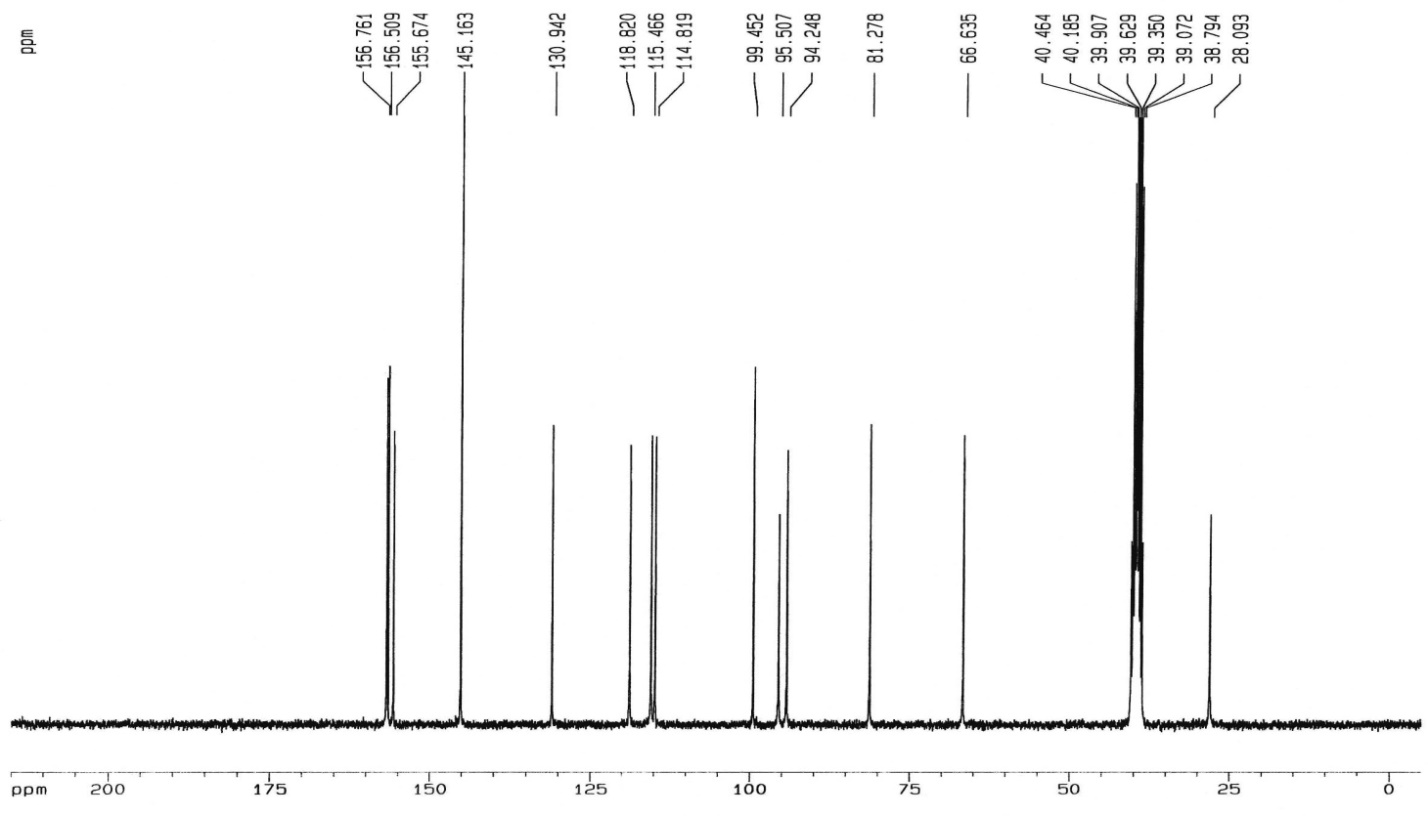


**(b)**

S2


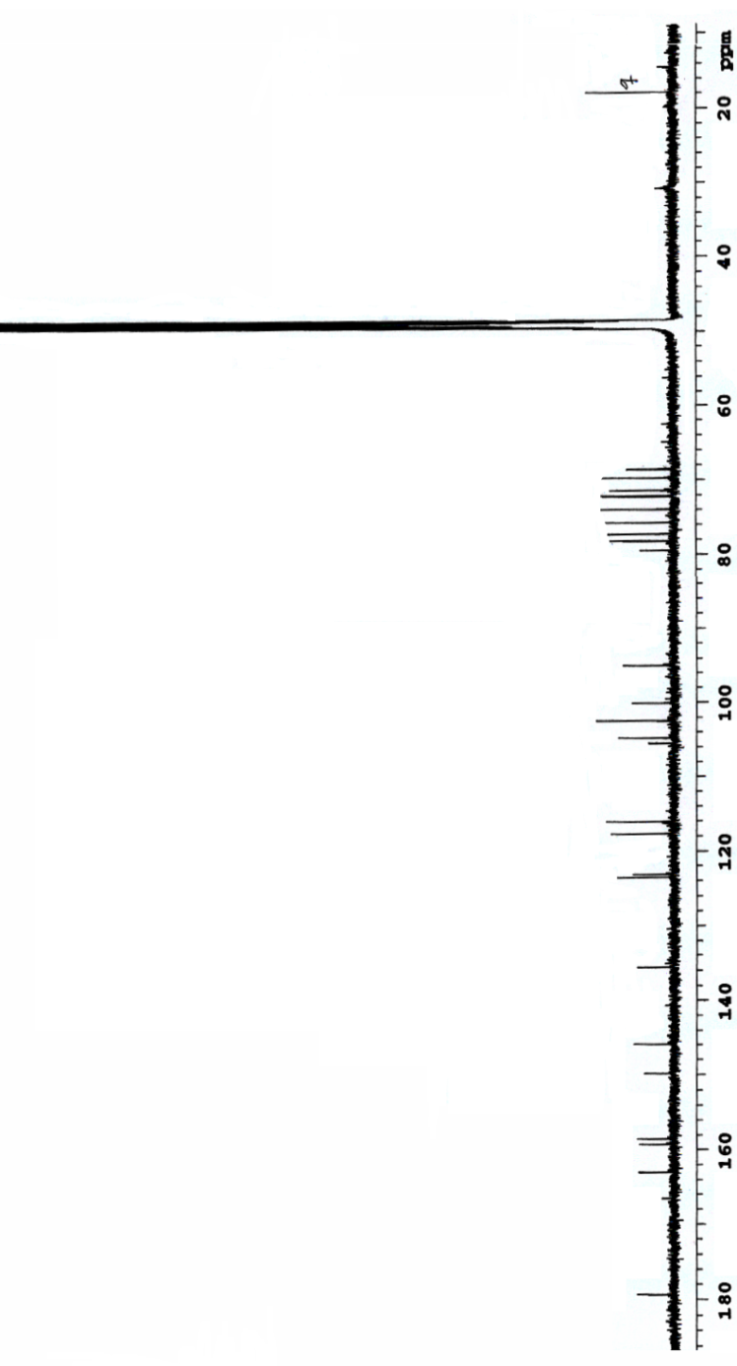

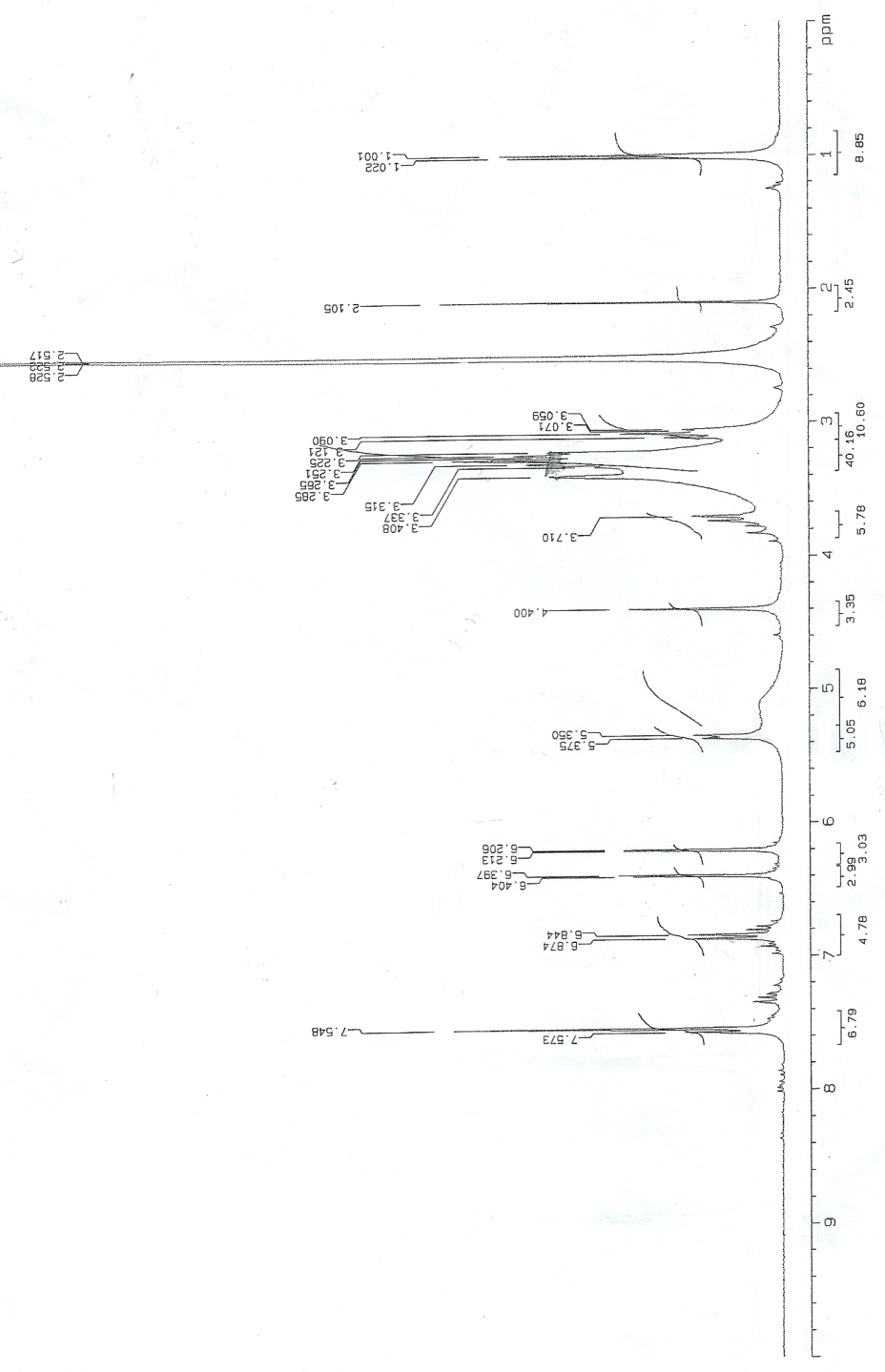


**(a)**

**(b)**

S3


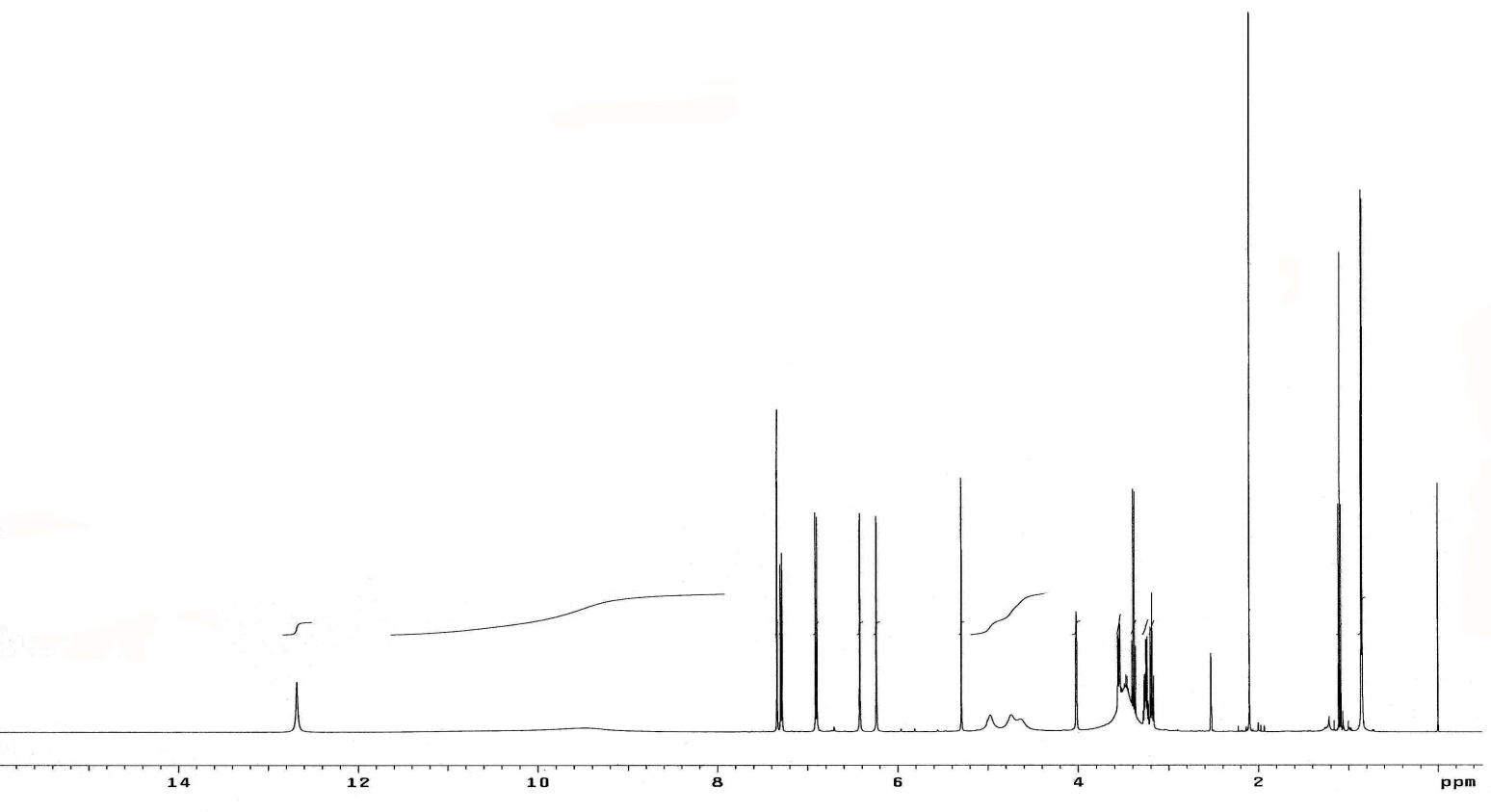


**(a)**


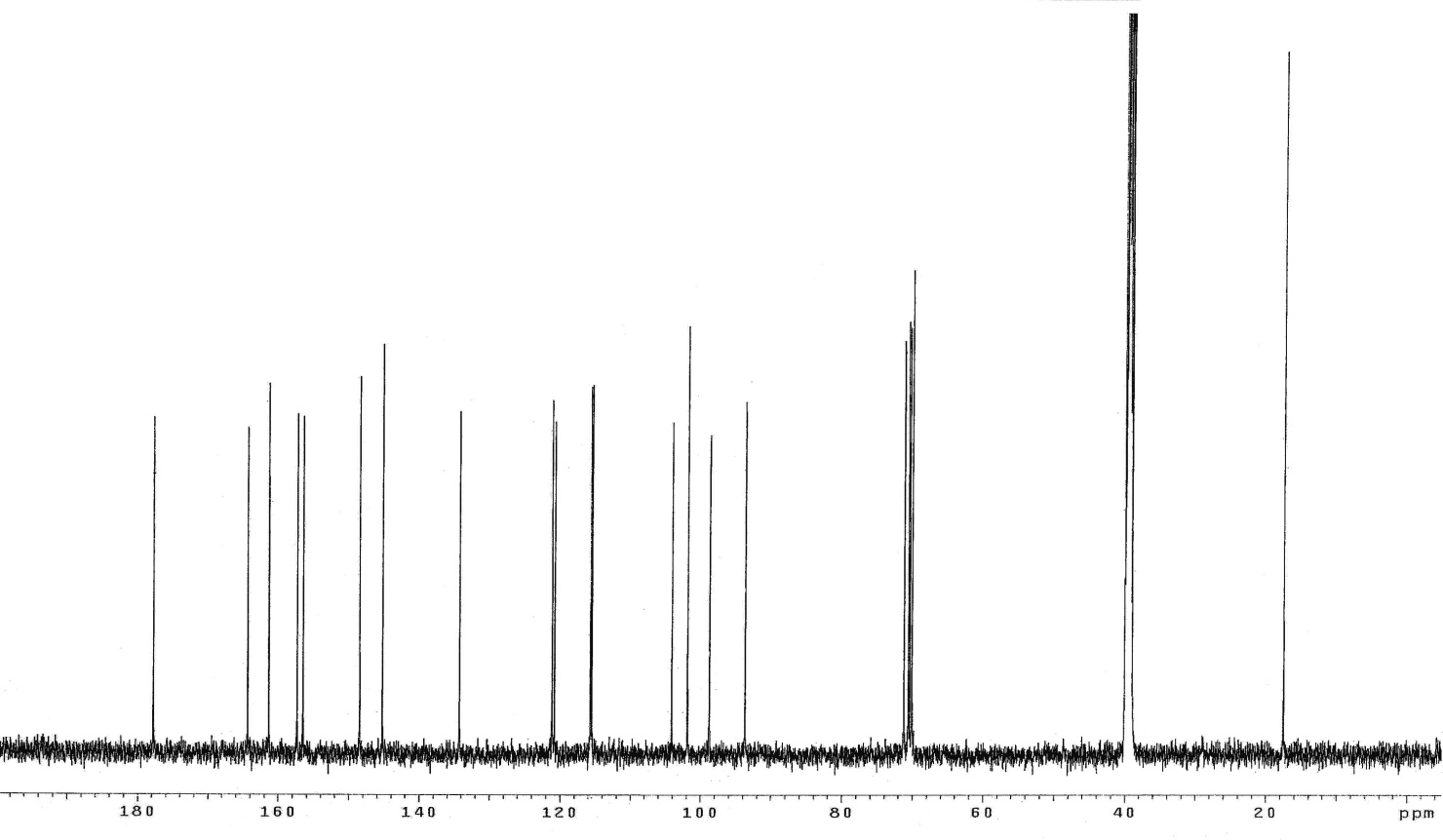


**(b)**

S4


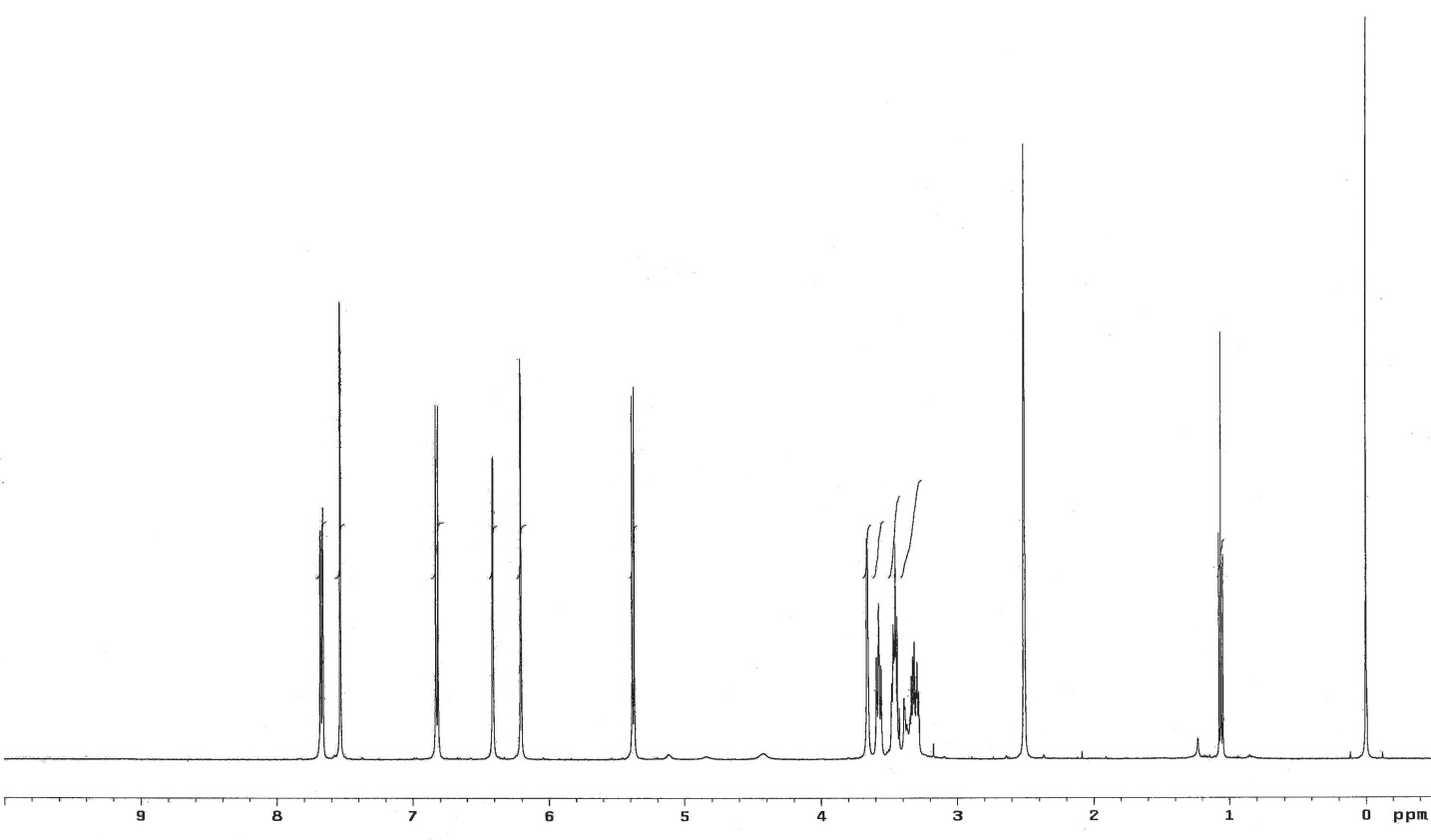


**(a)**


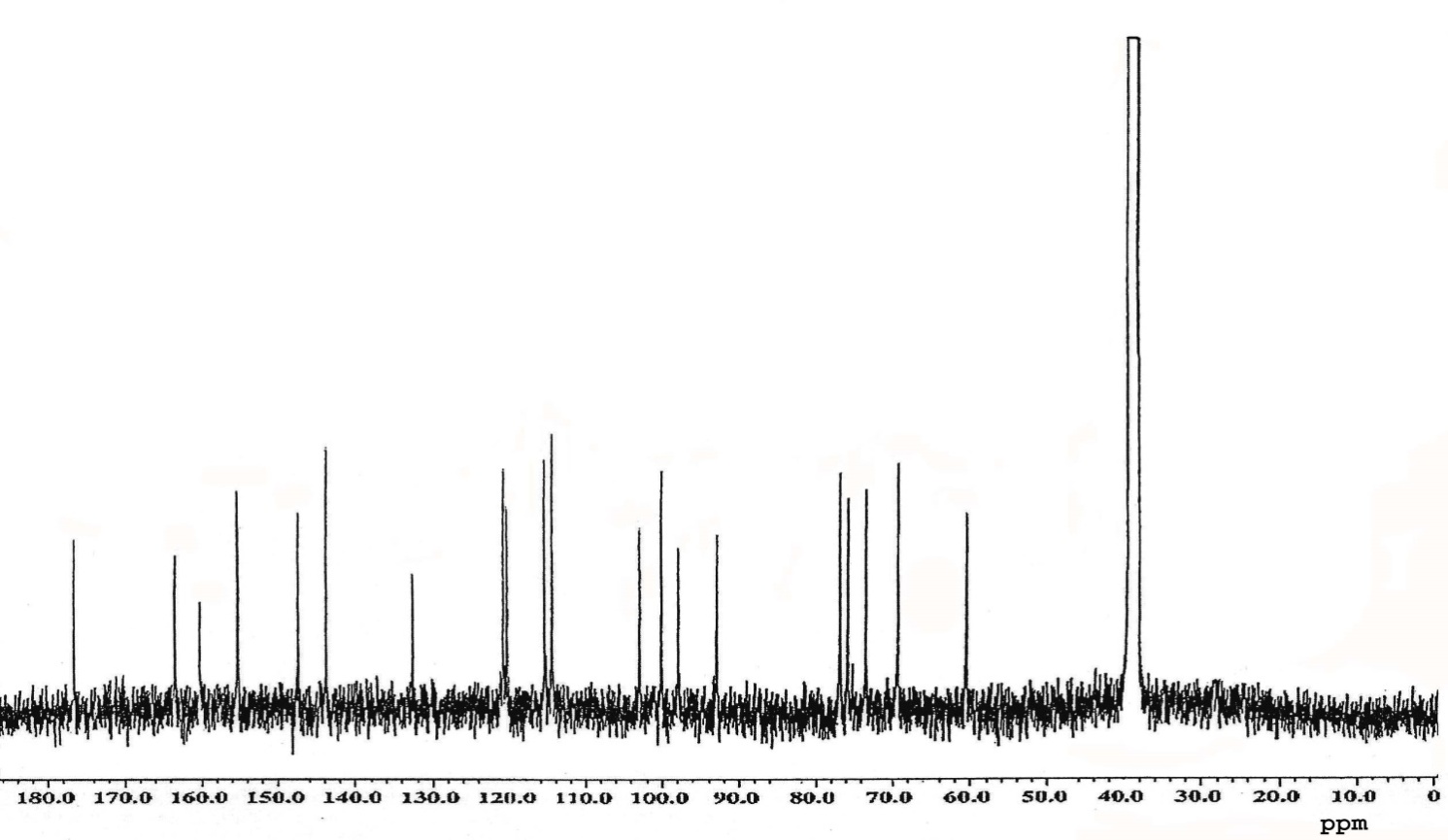


**(b)**

S5


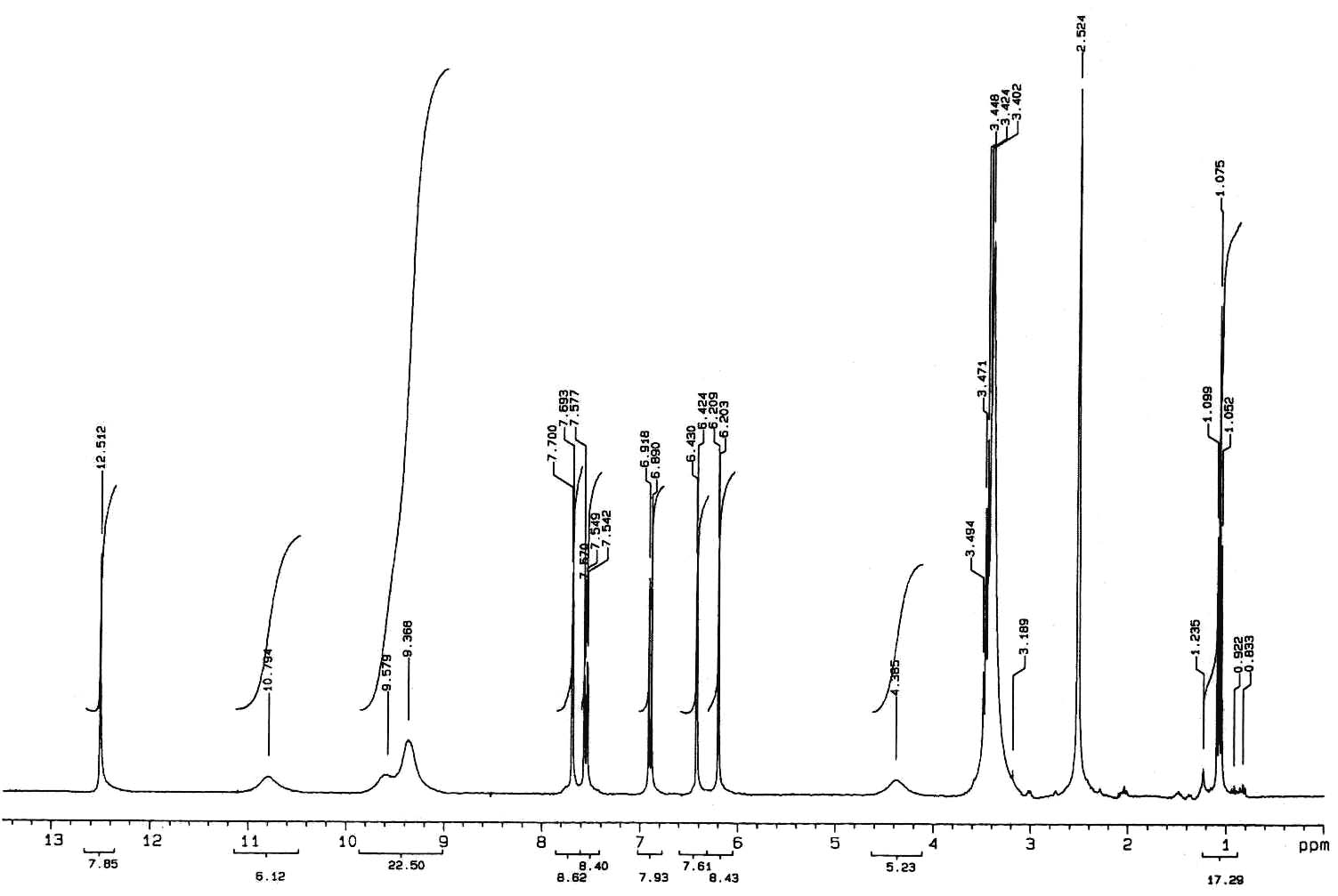


**(a)**


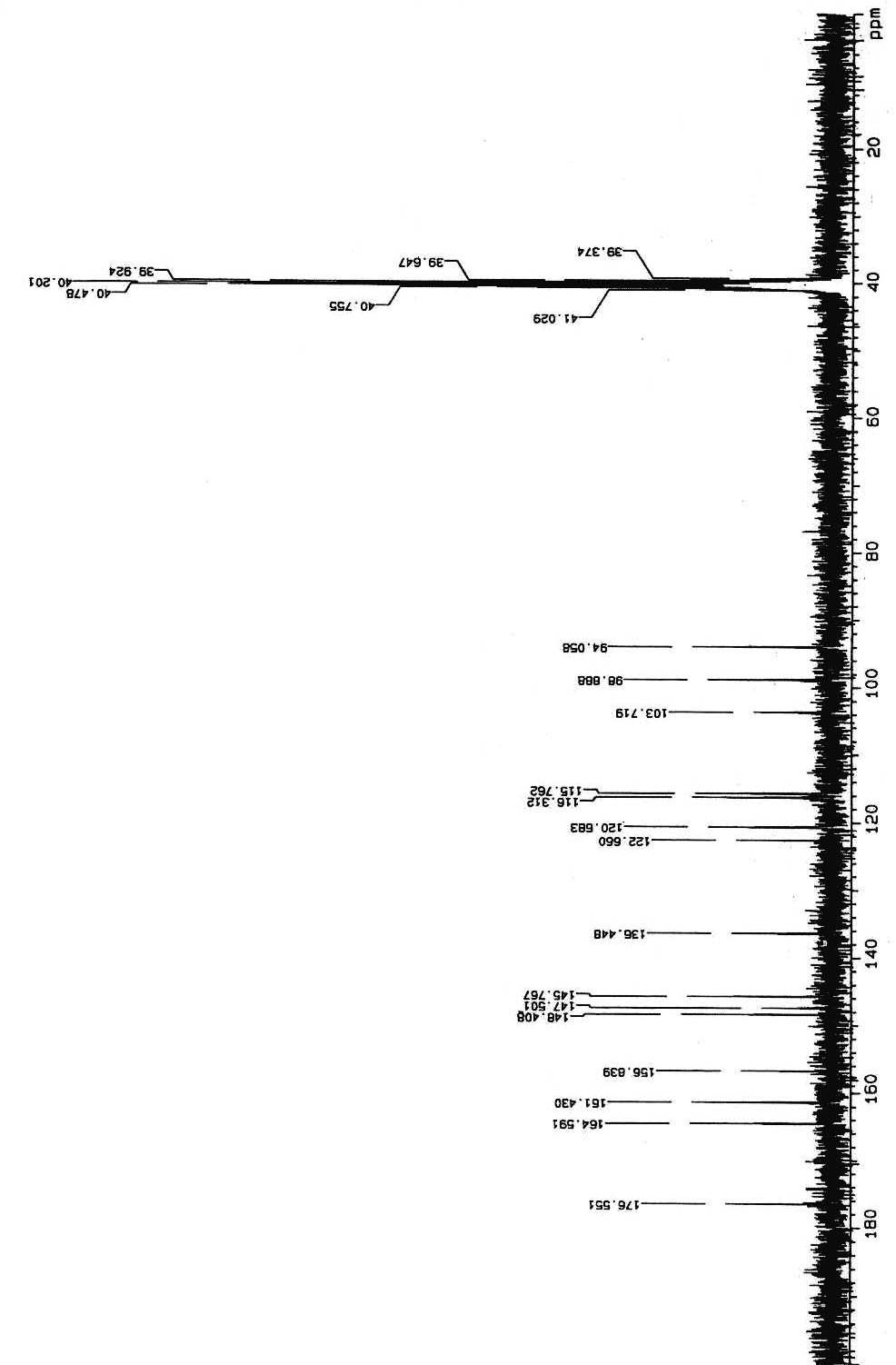


**(b)**

S6
